# Toward Humidity-Independent Sensitive and Fast Response Temperature Sensors Based on Reduced Graphene Oxide/Poly(vinyl alcohol) Nanocomposites

**DOI:** 10.1021/acsaelm.4c00729

**Published:** 2024-05-31

**Authors:** Ammar Al-Hamry, Yang Pan, Mahfujur Rahaman, Oleksandr Selyshchev, Christoph Tegenkamp, Dietrich R. T. Zahn, Igor A. Pašti, Olfa Kanoun

**Affiliations:** † Measurement and Sensor Technology, Chemnitz University of Technology, Reichenhainer Str. 70, 09126 Chemnitz, Germany; ‡ Semiconductor Physics, Chemnitz University of Technology, Reichenhainer Str. 70, 09126 Chemnitz, Germany; § Analysis of Solid Surfaces, Chemnitz University of Technology, Reichenhainer Str. 70, 09126 Chemnitz, Germany; ∥ Faculty of Physical Chemistry, University of Belgrade, Studentski trg 12-16, 11158 Belgrade, Serbia

**Keywords:** polymer−matrix composites, graphene oxide, poly(vinyl alcohol), nanocomposite, thin film, temperature sensor, humidity sensitivity

## Abstract

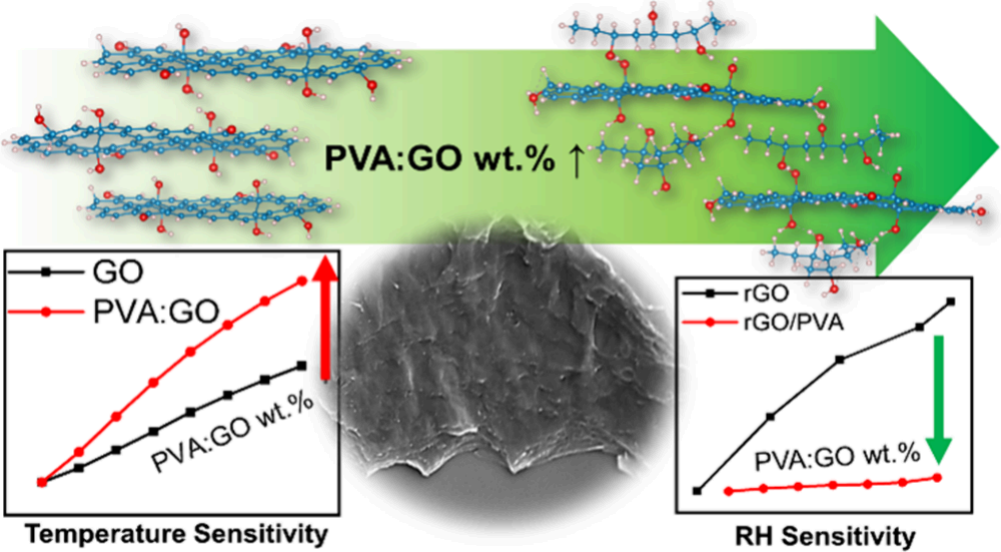

Flexible temperature sensors are becoming increasingly important these days. In this work, we explore graphene oxide (GO)/poly(vinyl alcohol) (PVA) nanocomposites for potential application in temperature sensors. The influence of the mixing ratio of both materials, the reduction temperature, and passivation on the sensing performance has been investigated. Various spectroscopic techniques revealed the composite structure and atomic composition. These were complemented by semiempirical quantum chemical calculations to investigate rGO and PVA interaction. Scanning electron and atomic force microscopy measurements were carried out to evaluate dispersion and coated film quality. The temperature sensitivity has been evaluated for several composite materials with different compositions in the range from 10 to 80 °C. The results show that a linear temperature behavior can be realized based on rGO/PVA composites with temperature coefficients of resistance (TCR) larger than 1.8% K^–1^ and a fast response time of 0.3 s with minimal hysteresis. Furthermore, humidity influence has been investigated in the range from 10% to 80%, and a minor effect is shown. Therefore, we can conclude that rGO/PVA composites have a high potential for excellent passivation-free, humidity-independent, sensitive, and fast response temperature sensors for various applications. The GO reduction is tunable, and PVA improves the rGO/PVA sensor performance by increasing the tunneling effect and band gap energy, consequently improving temperature sensitivity. Additionally, PVA exhibits minimal water absorption, reducing the humidity sensitivity. rGO/PVA maintains its temperature sensitivity during and after several mechanical deformations.

## Introduction

Flexible and thin-film sensors are becoming increasingly popular in wearable electronics and intelligent systems.^1−3^ They can be attached to the human body, clothing, artificial skins, or robotics to track body movements and continuously collect physiological data. Flexible temperature sensors with appropriate characteristics, such as sensitivity, flexibility, tensile properties, and stability, are required for these applications. Therefore, the development of sensor materials is essential to achieving these properties.

Nanomaterials are gaining popularity in developing high-performance sensors because they overcome the limitations associated with traditional materials such as metals, metal oxides, or ceramics^3,4^ concerning rigidity, conformity, and difficult assembling. In addition to the unique nanoscale advantages, such as high surface area, high surface-to-volume aspect ratio, and chemical functionalization, some of their key characteristics include compatibility with various substrates.^4^ In general, nanomaterials such as graphene, carbon nanotubes (CNTs), graphene oxide (GO), and metallic nanoparticles (NPs) are widely used for developing sensors for pressure and strain^5^ as well as temperature sensors.^6^ For temperature sensors, nanomaterial composites offer improved sensitivity, response time, and thermoelectric and mechanical properties. Combined with casting and printing techniques, they reduce fabrication steps, time, cost, and material waste in the deposition of sensing material compared to processes such as lithography and multistep coating.^7^ Temperature sensors have been fabricated using different materials, structure and fabrication techniques, layer-by-layer GO,^8^ electrospun poly(vinylidene fluoride) (PVDF)/silver NPs,^9^ laser direct writing of carbonized cellulose, GO and silver,^10−12^ inkjet-printed graphene/PEDOT:PSS,^13^ and screen printed graphene/BiFeO_3_.^14^ However, limitations with nanocomposites, such as homogeneity of dispersions and deposited or formulated structure, biocompatibility, biodegradability, and high integration, remain a challenge.^4,7^ Graphene oxide/poly(vinyl alcohol) (GO/PVA) composites are being studied because of their outstanding film-forming properties, adhesion qualities, and high tensile strength while remaining flexible.^15,16^ Thus, GO/PVA composite-based sensors have a promising performance and application potential. GO is a thin layer of carbon atoms produced by oxidizing graphite with strong oxidizing agents, followed by exfoliation.^17^ Because of the presence of large amounts of oxygen functionalities, GO can be easily dispersed in aqueous and polar organic solvents.^18,19^ In addition, GO can form stable dispersions and films via covalent or noncovalent interactions with polymers, nanoparticles, and other molecules.^19^ This is important to consider when combining materials with polymer matrices to improve their electrical and mechanical properties.^20^ PVA is a water-soluble synthetic hydrogel that has been used in the manufacturing of fibers, films, and gels.^16^ It is useful as a biomaterial for medical devices, as it is viscous and has a high water content. It is also nontoxic, biocompatible, and biodegradable. GO embedded in a PVA matrix in small amounts to form composites has been proposed in various forms, including nanocomposite films, hydrogels, and fibers. For instance, PVA/GO composite films were proposed to improve the tensile strength and Young’s modulus by increasing the weight percent of GO and have been applied for pressure sensors.^21^ In this regard, a freeze/thaw method was used to create a GO/PVA-based hydrogel.^16^ A colorimetric sensor for detecting ions was developed and investigated using GO quantum dots and PVA as hydrogel.^22^ Moreover, GO/PVA sensors have been investigated for humidity sensing using different principles. For instance, reduced graphene oxide (rGO) coated with tannic acid (rGO-TA) as a filler in PVA matrices demonstrated potential as humidity sensors with improved stability.^23^ GO quantum dots/PVA composite-coated optical fiber was developed as temperature and humidity sensors with a limited humidity range. The resulting temperature sensitivity was due to fiber temperature dependence.^24^ The use of GO/PVA as a humidity sensor in a redundant measurement approach with a micro-electromechanical system (MEMS) oscillator was demonstrated by employing the variation of its conductivity and mass, resulting in a change of the resonance frequency of the oscillator^25^ To the best of our knowledge, there are only very few publications on temperature sensors based on GO/PVA composites. Temperature sensitivity was observed in a GO/PVA hydrogel flexible strain/pressure sensor^26^ with high sensitivity and a temperature coefficient of resistance (TCR) of 3.5%. However, it has poor linearity and hysteresis behavior, while humidity influence is an important issue. These types of hydrogel sensors exhibit a volume phase transition temperature and are sensitive to mechanical and thermal deformation.^15^ Similarly, an ionic GO/PVA hydrogel was demonstrated as a dual temperature–force sensor, which was found to be modestly temperature sensitive in a small range of 30–45 °C.

Different materials, such as CNTs, graphene, and other NPs in a hybridized system or a polymer matrix, have been reported as temperature sensors.^27,28^ CNTs were used as temperature sensors using different fabrication configurations, such as vertically aligned CNTs grown on a substrate,^29^ CNT field effect transistors (CNTFET),^30^ and large-scale coated sensors.^31^ Several types of CNTs-based sensors were developed by printing and other coating techniques, such as drop, spin, and spray coating, where it seems that the deposition techniques have an insignificant influence on the sensitivity.^6^ Nevertheless, their performance as temperature sensors was quite limited. The TCR for CNTs is equal to or even lower than that of platinum thermistors, ∼0.3%/K, while the sensitivity is less than 0.1%/K in most cases.^6^ Single-walled nanotubes (SWNTs) deposited by dielectrophoresis were used in a cryogenic sensor at 2 K with a maximum TCR of ∼−1.5%/K,^32^ which is quite less at this temperature range. To increase the sensitivity of CNTs, they could be embedded in polymer matrices or hybridized with other materials. Nevertheless, big challenges are faced to homogeneously disperse the CNTs in a polymer matrix without changing their properties or leading to a lack of reproducibility. Several CNT composite sensors were demonstrated in the literature.^6,31^ MWNT/styrene-*b*-(ethylene-*co*-butylene)-*b*-styrene (SEBS), with 40 wt % loadings, shows in a small temperature range a TCR of ∼−4.4%/K.^33^ However, after several cycles, the TCR decreased to −0.06%/K. A high TCR of up to −10%/K was obtained by mixing MWNT with a phase-change hydrogel poly(*N*-isopropylacrylamide) (CNT-PNIPAm).^34^ The further disadvantage is that the TCR is highly humidity-dependent. Compared to CNT-based sensors, rGO was demonstrated to have higher sensitivity (TCR: ∼0.6345%/K) than MWNTs and SWNTs in the room temperature range.^35^ Graphene-based nanomaterials were shown to have a high sensitivity of up to 3%/K for graphene platelets in a limited linear range of 10–60 °C.^36^ However, poor repeatability and hysteresis behavior are observed. Different forms of graphene and its derivatives, particularly GO and rGO, provide various sensing possibilities.^37^ The sensitivity commonly reported for graphene-based sensors ranges from 0.06%/K to greater than 2%/K.^13,35^ Similarly to CNTs/polymer composites, graphene nanowalls/PDMS was found to have a positive TCR of 20%/K in a limited range of 35–45 °C,^38^ which is attributed to the thermal expansion of the polymer substrate, which makes them as well sensitive tactile sensors. Even though such composite sensors with an elastomer polymer can reach high TCR values, they are generally limited in the temperature range. In addition, the temperature sensitivity originates from thermal expansion, indicating mechanical deformation and humidity sensitivity^39^ where the selection of polymer and nanofiller will determine the behavior of the sensor with either a positive or a negative temperature coefficient (PTC or NTC). Furthermore, the multifunctional response of nanocomposite sensors makes it challenging to separate the signals corresponding to certain stimuli.^7,40^

Sensors based on polymer nanocomposites are sensitive to humidity due to the existence of functional groups and the different degrees of hydrophilicity of sensor surfaces. For example, GO and rGO experience a change over time in the resistance because of exposure to air or degradation because of humidity.^41^ To overcome that, sensor encapsulation was used to protect the sensor from long exposure to moisture.^7^ In this regard, several works have been proposed. Rehman et al. have coated the PEDOT:PSS thin film temperature sensor on a glass substrate using Al_2_O_3_ by atomic deposition which was proved to optimize the performance of the sensor even under water.^42^ Kim et al. have developed a PVA-based temperature sensor with Al_2_O_3_ encapsulation which performs better than a nonencapsulated one.^43^ Both works concluded the encapsulation serves for the elongation of lifetime. For a flexible substrate, the coated Al_2_O_3_ layer and graphene/Al_2_O_3_ on poly(ethylene naphthalate) (PEN) achieve a good water vapor transmission rate with rather crack formation upon bending.^44^ For the flexible temperature sensor, as an example it was reported that silicon paste was used for passivation of the CNT-based fiber sensor with a thickness limiting the performance, i.e., flexibility.^45^ However, the effect of the humidity was not reported. In another paper, polyimide (PI) type was used to encapsulate the rGO temperature sensor to prove stable resistance change at high humidity, with no comparison of the sensor before and after passivation.^11^ In general, challenges in the encapsulation of flexible electronics are still found such as heat dissipation and those related to nonplanar flexible devices.^46^ Temperature sensors with humidity independence were demonstrated where layer-by-layer positively/negatively charged was deposited on fiber and reduced chemically to show low response at high humidity.^8^ Jung et al. coated rGO-based films with PI, silicon, and GO to prove GO-PI demonstrated stability and humidity dependency.^41^

Based on the discussion above, this paper investigates the potential for the development of GO/PVA composite films, where compared to the literature high mixing percentages of the initial GO/PVA composites are employed. The goal is to improve the characteristics of rGO as a temperature sensor while preserving the inherent temperature conductivity mechanism of rGO as a semiconducting material. rGO has a very good temperature sensitivity when reduced at relatively low temperatures. It has semiconducting temperature dependence behavior; i.e., it has a negative temperature coefficient. However, it has been shown to have stable properties when annealed at higher temperatures at the cost of lower sensitivity.^47^ The trade-off between good sensitivity and stability is therefore important. Reduction at such low temperatures is chemical-free, environmentally friendly, and energy-saving and does not require expensive resources, e.g., furnaces or reducing agents. GO/PVA significantly increases both stability and sensitivity compared to that of rGO alone and acts as an insulating layer. Mixing PVA in a high-weight proportion with GO significantly is expected to lower the influence of humidity. Films on flexible substrates can be realized with such techniques toward humidity-independent temperature sensors to avoid the use of a passivation layer, which increases the thickness for critical applications such as embedding in wearable and energy devices, e.g., batteries.

The effect of various reduction temperatures on the obtained resistance range of rGO and rGO/PVA and their sensitivity to temperature and humidity are explored in this paper. To compare the effect of humidity of bare rGO/PVA, encapsulated rGO/PVA films with PDMS and PI layers were made, which can withstand higher humidity (90%). Briefly, the temperature sensitivity for several films reduced at different temperatures was examined in the range from 10 to 80 °C. In addition, temperature cycling (10–80 °C) and relative humidity measurements (10–80% RH) were performed to determine the best performance attained by altering the mixture ratio and reducing temperature. Scanning electron microscopy, atomic force microscopy, ultraviolet–visible–near-infrared (UV–vis–NIR), Raman, and X-ray photoemission spectroscopy (XPS) were used to investigate the physical and optical characteristics of GO and GO/PVA. In addition, several experiments were carried out to leverage the sensor usability and features in various application setups. Furthermore, the mechanical properties of the sensor were tested by performing bending cycles, and temperature sensitivity was measured. Temperature sensitivity of the bent film under different curvatures was also carried out. Moreover, reproducability, repeatability and stability of the rGO/PVA sensros were also investigated.

## Experimental Investigations

### Materials and Preparation

GO (SKU-HCGO-W-175) was obtained from Graphene Laboratories Inc. (Ronkonkoma, NY) as a water dispersion with a nominal concentration of 5 g/L. It was produced chemically using the Hummers method with flake sizes of 0.5–5 μm. Approximately 60% of the flakes have an atomic thickness. The concentration of the GO solution was determined to be 0.39 wt % after a gravimetric check in a crucible cup.

PVA powder (Sigma-Aldrich; CAS Number 9002-89-5; *M*_w_ 85,000–124,000) was mixed with water to make a 2 wt % aqueous solution. The PVA preparation procedure was carried out as follows. Deionized water (Diwater) was first added to the glass vial and then placed on a magnetic stirrer and heated to 90 °C. A Pt100 temperature sensor was installed to monitor the water temperature. When the water temperature reached 90 °C, the rotation rate was increased to 800–1000 rpm, and the weighed PVA powder was slowly added to the water until PVA was completely dissolved. The temperature was reduced to room temperature and kept constant, and the mixture was stirred for a further 2 h.

**Figure 1:**
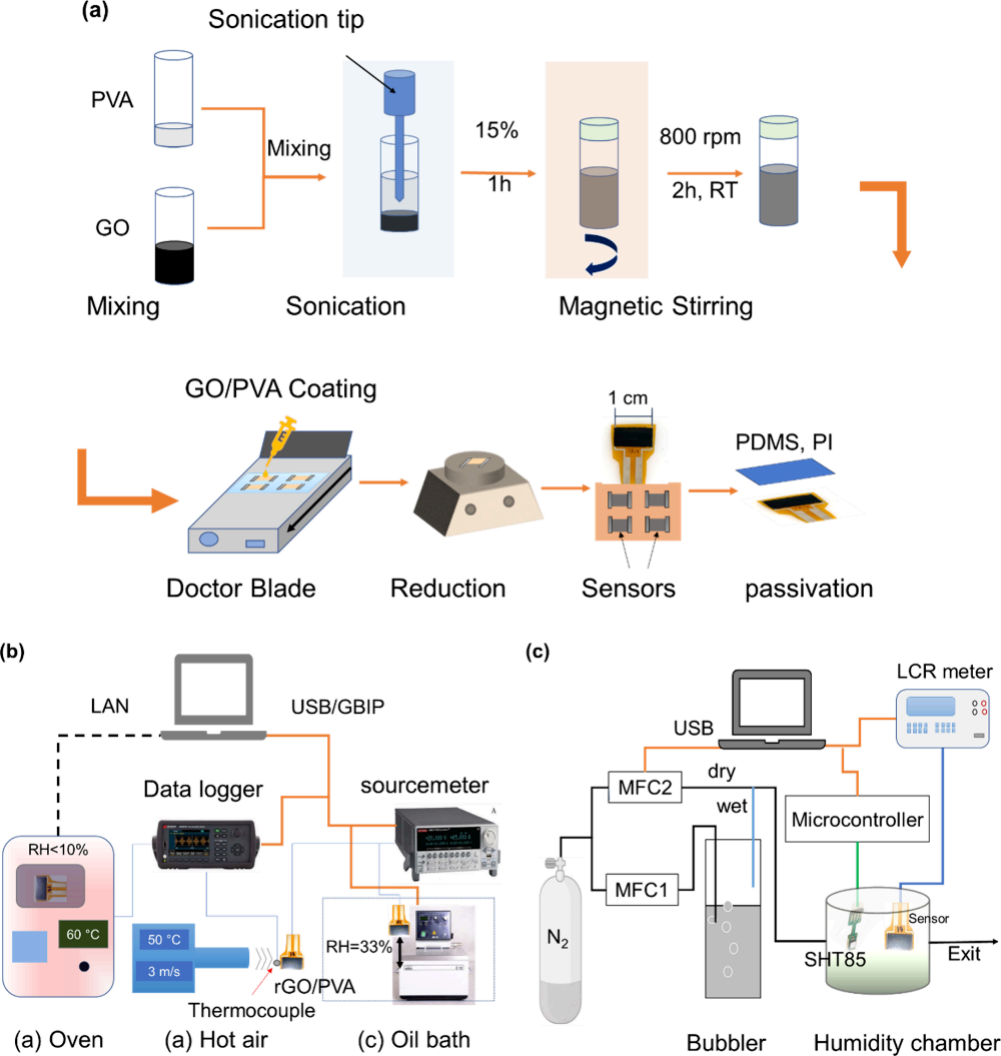
(a) Sensor materials preparation and film coating, (b) temperature measurement setup, and (c) setup for humidity measurement.

Different PVA solution volumes were mixed with the GO aqueous solution (0.3 wt %) to obtain different GO/PVA weight ratios (GO/PVA w/w %). As shown in Figure 1a, each mixture was sonicated for 1 h at 15% sonication power of 200 W (GM 3200, Bandelin electronic, Berlin, Germany), followed by 2 h of 800 rpm stirring. The films were prepared on a PI substrate (Kapton HN, Dupont) with the doctor blade technique (ZUA 2000 universal applicator, Zehntner Instruments, Switzerland) using the same amount, time, and coating speed. To achieve stable deposition, the films were prepared by setting the gap height of the wiper on the ZUA 2000 machine to 420 μm. The reduction of the film was carried out using a hot plate at different temperatures. The passivation for some sensors was performed by coating PDMS or using PI tape while the sensors are on the hot plate at 80 °C.

### Temperature and Humidity Measurement

The characterization process of temperature and humidity is shown in Figure 1b,c. The temperature characterization was carried out using a VT 2002 temperature test chamber VT 2002 (Weiss Technik, Germany) in the 10–80 °C range. Electrical measurements were performed by a DAQ973A data acquisition system (Keysight, USA). For humidity tests, films were measured at various humidities (10–80% RH) generated by two gas flows of dry and wet nitrogen controlled by two mass flow controllers EL-FLOW Prestige (Bronkhorst High-Tech B.V., Netherland), regulated by a LabVIEW program. The sealed humidity chamber was equipped with a calibrated SHT85 humidity and temperature sensor from (Sensorion GA, Switzerland) to monitor the conditions within the chamber. Temperature-dependent current–voltage (*I*–*V*) measurements (−1 to 1 V) were carried out by a Keithly 2602 sourcemeter. For rGO/PVA, low-temperature measurements were carried out in a vacuum chamber (0.1 mbar) cooled by liquid nitrogen in the range of ca. 100–350 K. The experiment of dipping the sensor in hot rotating oil was carried out by plunging the sensor into a temperature control to get an abrupt change between the room temperature and the hot oil bath. In addition, response time by exposure to hot air (speed of 3 m/s after DIN EN IEC 60751:2023-06) was measured for uncoated and coated sensors, and the sourcemeter registered the resistance against time. The cyclic bending test was carried out by a setup developed for this purpose based on step motor control by a microcontroller to obtain a maximum bending angle of ∼50°. Strain testing was performed by an Instron ElectroPuls E10000 universal testing machine (UTM) in the range of 0–1% strain, and resistance was measured by the data logger. Information about physical characterization tools and semiempirical quantum chemistry calculations are given in the Supporting Information, Sections S1.1 and S1.2, respectively.

## Results and Discussion

### Physical Characterization of the GO/PVA Composites

**Figure 2:**
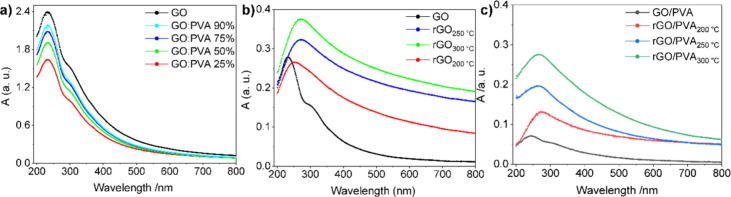
UV–vis–NIR absorption for GO and GO/PVA composites: (a) GO and GO/PVA dispersions, (b) GO and rGO, and (c) GO/PVA-based films.

The UV–vis spectra of the GO and GO/PVA dispersions (Figure 2) were collected after the dispersions were diluted 50 times in water. The absorption peak in the GO film spectra at around 230 nm indicates the π–π* transition of the C–C bond. At 300 nm a shoulder which indicates the n−π* plasmonic transition of C=O bonds is noticed.^48^ The existence of the shoulder at ∼300 nm as representative of GO for GO/PVA is evidence of good dispersions. As expected for carbon nanomaterials, the spectra decline with an increasing wavelength. As shown in Figure 2a, the GO absorption peak at 230 nm diminishes as the GO concentration decreases in GO/PVA dispersions. Figures 2b and 2c depict respectively the untreated and temperature-reduced (200, 250, and 300 °C) transparent GO and GO/PVA-50% spin-coated films on glass substrates. Nonreduced films possess the same characteristics as GO and GO/PVA dispersions. While the films were reduced, the shoulder vanished, suggesting that reduction occurred for all temperatures. In addition, a red-shift of the peak from 230 to 250–270 nm is seen as a notable indicator of GO reduction to rGO. It increases gradually by increasing the reduction temperature and thus the reduction degree.^48^ Moreover, by increasing the reduction temperature, the absorbance of rGO and rGO/PVA films increases as they become darker than brownish-transparent GO. In Figure S2, the optical band gap energies were determined by using the Tauc plots extracted from the UV–vis spectra. The band gaps of reduced GO films range from 3.5 to 3.7 eV for pristine GO to 1.9, 1.44, and 1.38 eV for GO reduced at 200, 250, and 300 °C, respectively. For rGO/PVA the band gaps are estimated to be 2.1, 1.96, and 1.87 eV for GO/PVA reduced at 200, 250, and 300 °C, respectively. These results confirm that the composites have finite band gaps, which are higher than those of neat rGO films.

**Figure 3:**
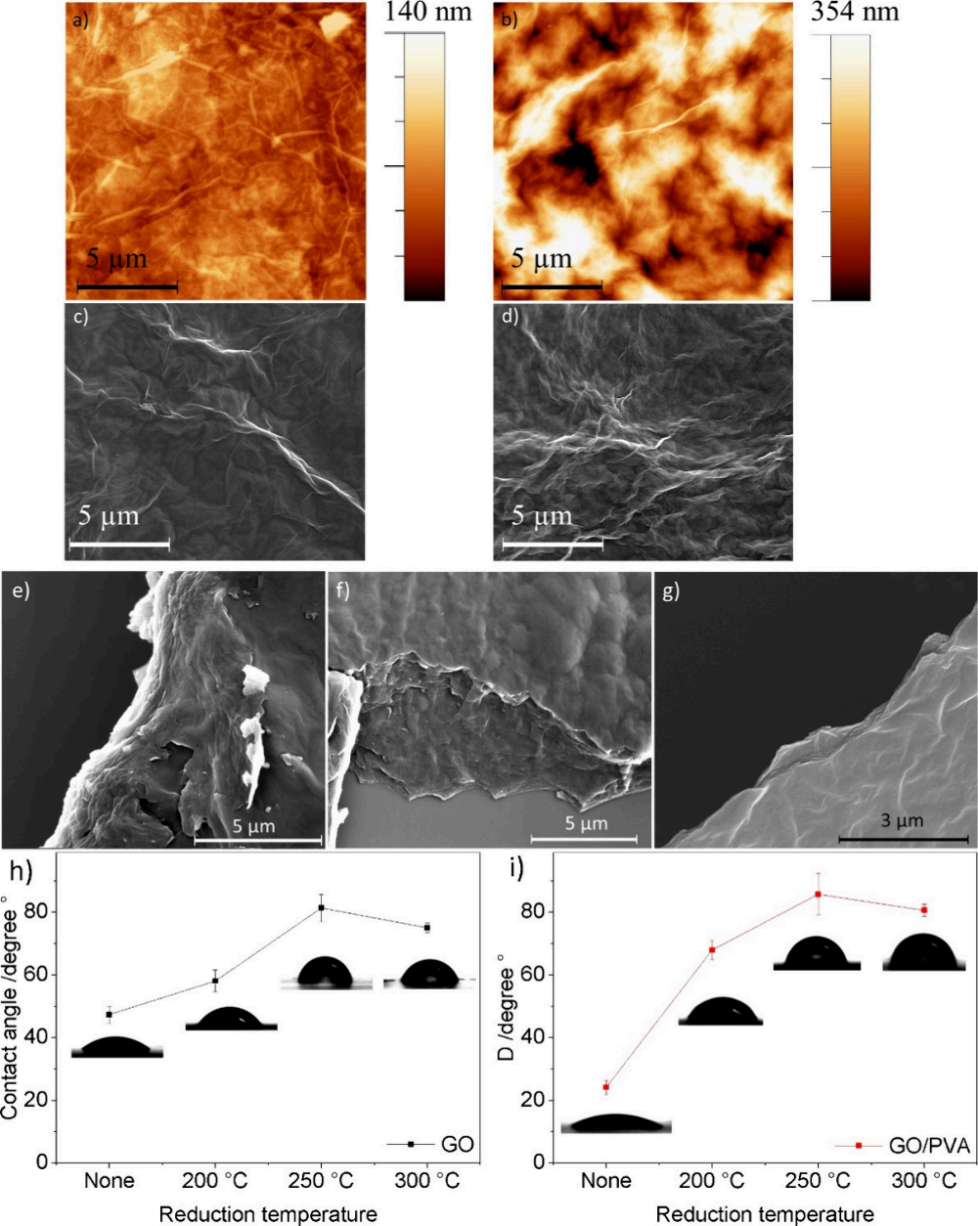
AFM images of (a) rGO and (b) rGO:PVA 50%, SEM images for (c) rGO and (d) rGO:PVA 50%, and SEM images showing the differences of layered edges of (e) rGO:PVA 25%, (f) rGO:PVA 75%, and (g) rGO. (h) and (i) are the contact angle evolution by reduction temperature for GO and GO/PVA, respectively.

Figure 3a–d shows the AFM and SEM images of rGO and rGO/PVA 50%. Both samples were drop-casted on silicon wafers and thermally reduced at 250 °C. Both SEM and AFM confirm the excellent dispersion of the composites. However, aggregates appear due to the drop-casting deposition process. AFM gives information about the roughness, which is higher for rGO/PVA composites than in the case of rGO. Typically, for high-concentration drop-casted dispersions, roughness is expected to be high, and single features cannot be observed.^49^

To estimate the GO flake size and distribution, the films were prepared by drop casting using as-received GO and after sonication. In both cases, 20× dilution was necessary to image individual flakes. In Figure S3a, the roughness is much smaller than that in Figure 3, where the roughness is only 20 nm; single flakes of ∼1 nm thickness can be seen clearly. However, the flakes are mostly stacked and intersected. In Figure S3b, for the sonicated dispersion, the flakes are much smaller laterally. The morphology of GO after sonication indicates the breaking of the flakes into smaller pieces. In addition, two samples of GO and GO/PVA spin-coated on silicon substrates were imaged (see Figure S4). Even at different scales of the scanning, the coating is ultraflat with full coverage, high homogeneity, and very low roughness, less than 6.5 nm for a 30 μm^2^ scanning area. The images of GO/PVA also reveal excellent uniformity of the film, with moderate roughness of a maximum of 12 nm. For different GO and GO/PVA samples, SEM images of drop-casted samples indicate aggregation as a dominating feature, which is due to the nature of the drop-casting procedure (see Figure S5). In addition, the GO structure appears compared to GO/PVA, which seems to be opaque due to the PVA insulating nature. This is also noticeable by comparing both AFM and SEM images in Figure 3a–d, confirming the consistency between the AFM and SEM images.

Most importantly, the SEM images in Figure 3e–g indicate the layered structure of rGO/PVA and rGO, showing rGO flakes stacking with PVA embedded between them. These images were taken at broken edges, where the chance to reveal such formation is high compared to that in intact film areas. The white color on the edges in Figures 3e and 3f indicates the high content of PVA separating the 2D layers of GO, more apparently for rGO:PVA 25%. Additionally, the SEM edge images (see Figure 3e–g), combined with the AFM results (see Figure S3), confirm that even for drop-cast samples, the rGO flakes form well-arranged stacked layers regardless of the surface topography and roughness. The significant difference between the images corresponds to the decrease of PVA content and hence insulation gap between the rGO layers. This explains the sensitivity to the temperature where the insulating PVA gaps between the flakes play a crucial role as the electron transport would require higher activation energy.^50^ Contact angle measurements were conducted to establish the surface wettabilities of GO and GO/PVA. Figures 3h and 3i illustrate these measurements before and after the reduction process. Initially, the contact angle for GO was approximately 47.34 ± 2.7°. The contact angle increased upon thermal reduction and reached a maximum value of 81.35 ± 4.33° at a reduction temperature of 250 °C. The increase in the reduction temperature resulted in a higher hydrophobicity of the surface due to the loss of oxygen and hydrogen groups. When the temperature is further increased, reduction leads to the generation of small cracks and holes on the surface caused by degassing due to heating. As a result, the contact angle for rGO300 °C slightly decreases. These trends apply similarly to rGO/PVA composites. In addition, the contact angle for nonreduced GO/PVA is even smaller compared to GO. However, for reduced films of rGO/PVA, the contact angles obtained are higher than those of rGO, 85.72 ± 6.69° for rGO/PVA_250 °C_, due to the contribution of rGO and also the baking of PVA.^51^

**Figure 4:**
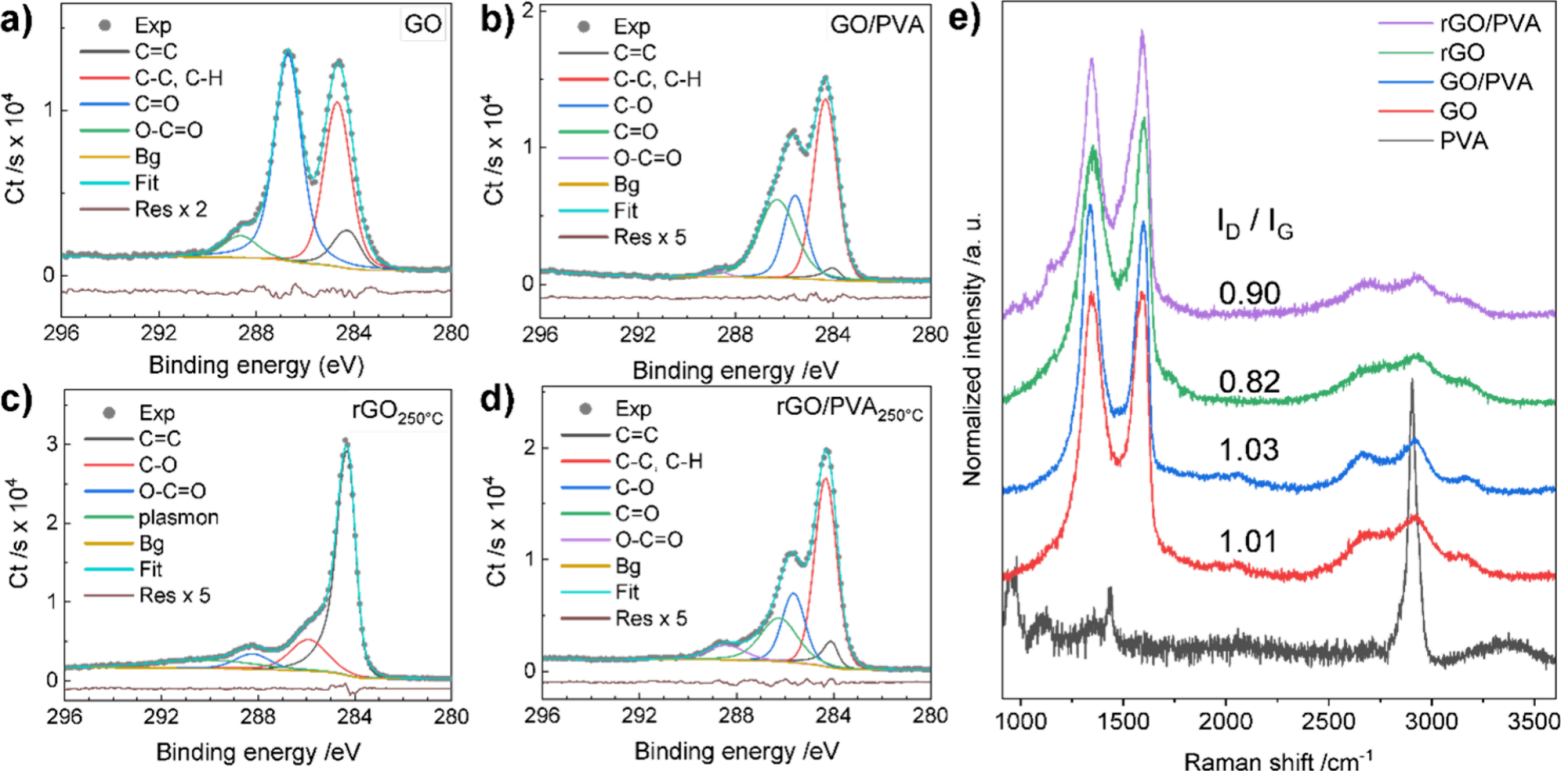
C 1s high-resolution XPS spectra of (a) GO and (b) rGO reduced at 250 °C, (c) GO/PVA, and (d) rGO/PVA reduced at 250 °C. (e) Raman spectra of PVA, GO, and GO/PVA before and after reduction at 250 °C.

XPS was used to investigate GO and GO/PVA composites before and after thermal reduction. Four components can be identified in the C 1s carbon peaks of the initial GO samples’ XPS spectrum (Figure 4a). The peaks at (284.2 ± 0.1), (284.8 ± 0.1), (286.7 ± 0.1), and (288.5 ± 0.1) eV correspond to C=C in aromatic rings, C–C defects, C=O, and O–C=O bonds, respectively; these moieties are typical for GO.^49^ The spectrum of GO/PVA (Figure 4b) shows functional groups as in GO, revealed by fitting at (284.0 ± 0.1), (284.3 ± 0.1), (286.3 ± 0.1), and (288.7 ± 0.1) eV. Additionally, it shows a significant contribution of C–O bonds at (285.6 ± 0.1) eV, indicating the presence of hydroxyl groups of poly(vinyl alcohol). The appearance of the C–C component at a binding energy (284.3 ± 0.1) eV, lower than expected at (284.6–285.0 eV), is mainly due to the contribution of poly(vinyl alcohol). The C 1s peak of PVA with a high molecular weight was previously reported at 283.8 eV.^52^ The significant suppression of the carbon–oxygen-related C 1s peaks at 286.5–288.5 eV in the thermally reduced GO sample (see Figure 4c) confirms that oxygen groups are significantly removed at 250 °C. The observed narrowing of the C 1s peak and the appearance of a π–π* satellite shoulder at (290.2 ± 0.1) eV indicate an increase in C=C graphitic bonds. Because of the noticeable conductivity of rGO, the C 1s peak is usually fitted by an asymmetrical function.^49^ The situation is different for rGO/PVA, where the C=O peak is partially suppressed, but the C–O peak is still prominent and the relative intensity of the O–C=O peak increases (see Figure 4d). This behavior can be explained by the reduction of the GO component (elimination of the C=O groups) while the PVA part remains unchanged. The C/O ratios for GO, GO/PVA, rGO, and rGO/PVA, calculated using C 1s and O 1s spectra are 2.0/2.2/4.3/3.1, respectively (averaged over several areas on the sample surface). No significant decomposition of PVA at 190 °C was observed,^53^ as confirmed here by XPS, which shows abundant oxygen-related groups in the 285.7–286.2 eV binding energy range.

Furthermore, Raman spectroscopy is performed to study the effects of thermal treatments on GO and GO/PVA films. The Raman spectra of as-deposited GO, GO/PVA, and thermally reduced rGO and rGO/PVA (50 wt %) at 250 °C are shown in Figure 4e. The most pronounced peaks at around 1350 and 1580 cm^–1^ are assigned to the D and G bands, respectively. The G peak corresponds to the E_2g_ phonon mode at the center of the Brillouin zone. The D peak is the breathing mode of the carbon hexagonal ring and only becomes Raman active with the existence of defects.^54^ The features at higher frequencies (from 2500 to 3200 cm^–1^) are second-order bands. The peak at around 2700 cm^–1^ is usually assigned as the 2D band, which originates from a double resonance effect. The overtone of the G band is located at around 3200 cm^–1^, which is termed the 2G band, while the peak at around 2940 cm^–1^ is a combination of the D and G, which is usually assigned as D + G.^55^ As mentioned above, the D band is only active with the presence of defects, which makes the intensity ratio *I*_D_/*I*_G_ sensitive to the defect density and, consequently, the degree of reduction.^56^ As shown in Figure 4e, *I*_D_/*I*_G_ values are calculated to be 1.01 and 1.03 for GO and GO/PVA and 0.82 and 0.90 for rGO and rGO/PVA, which indicate the large defect density and increased reduction after thermal treatment at 250 °C, which is in agreement with XPS results. Based on the change of the *I*_D_/*I*_G_ ratios upon reduction and the position of the G band (∼1590 cm^–1^), rGO in all the samples is in the first stage of the amorphization trajectory,^57^ indicating a restored sp^2^ system and consequently conductivity.

The semiempirical calculations performed for PVA–rGO and (PVA)2–rGO model complexes (Figure S6) suggest that rGO and PVA interact via noncovalent interactions while the formation of hydrogen bonds between −OH groups of PVA and −OH of rGO was visible. The model with one PVA fragment and one rGO flake has a HOMO–LUMO gap of 4.17 eV, slightly less than that of pure rGO. However, upon adding more PVA and building the (PVA)2-rGO model, the HOMO–LUMO gap increases to 4.29 eV. Thus, one could expect that the resistance of the rGO/PVA films increases with increasing PVA content, confirmed experimentally, as described in the next section. Moreover, the HOMO–LUMO gap of the system is determined mainly by the rGO. Thus, the temperature dependence of the resistance is also likely to be determined by the rGO component.

### Electrical Characterization of the GO/PVA Composites

**Figure 5:**
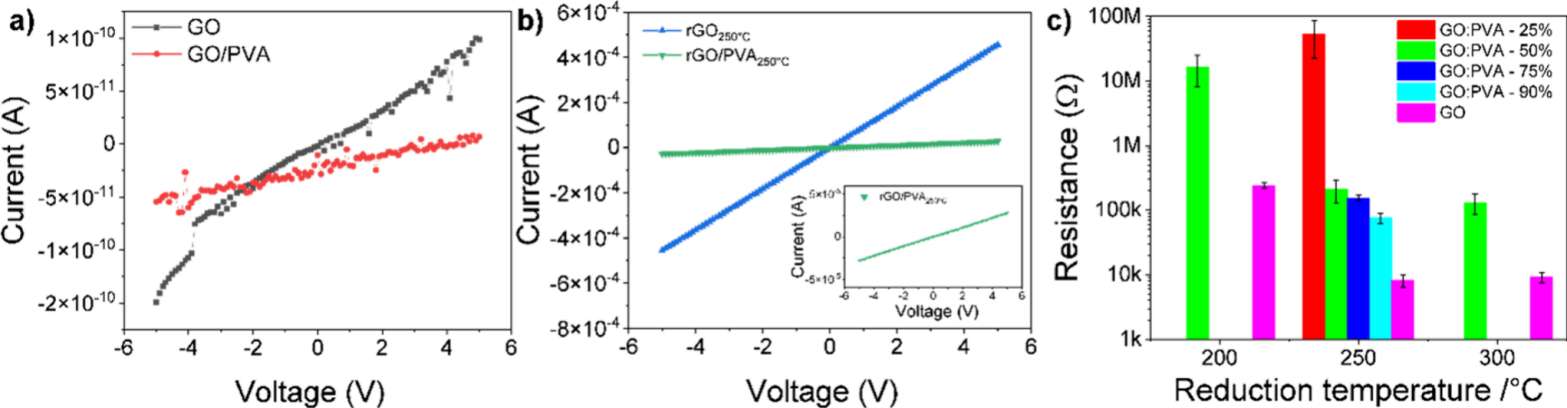
*I*–*V* curves measured by a sourcemeter for (a) nonreduced GO, GO/PVA prepared using 2% PVA solution and having a 50% mixing ratio; (b) the same material after reduction at 250 °C; and (c) electrical resistance dependence on different preparation parameters, i.e., the effect of the GO:PVA mixing ratio and the effect of the reduction temperature.

Following the coating process, the electrical resistance of the GO and GO/PVA films was measured by the sourcemeter and was in the range of hundreds of GΩ. Because of the dominant sp^3^ hybridization, these are practically electrically insulating films. In addition, the GO/PVA composites become more insulating due to the presence of PVA. Figure 5a shows the *I*–*V* curves for GO and GO/PVA at a 50% mixing ratio. Then, the samples were placed on a hot plate for conventional thermal treatment to reduce GO and, thus, enable conductive electrical properties. In Figure 5b, the *I*–*V* curves are shown for rGO and rGO/PVA reduced at 250 °C for comparison with those of the nonreduced samples. The resistance decreased several orders of magnitude, from hundreds of GΩ, corresponding to electrically insulating materials, to 11 kΩ for rGO and 178 kΩ for rGO/PVA, to form an ohmic resistance. This result confirms that the temperature treatment at 250 °C is adequate to obtain a considerable increase and thus easily measurable conductivity by any usual voltmeter device.

GO and GO/PVA (GO:PVA 50%) were also reduced at 200, 250, and 300 °C. The resistances of rGO and rGO/PVA composites decrease as the reduction temperature increases due to the efficient reduction and restoration of the graphene-like structure in rGO.^49^ The rGO films always had a lower resistance than rGO/PVA reduced at the same temperature. Beyond 200 °C, there is a sharp decrease in the resistance. The effect of reduction is observed as a decline in the electrical resistance. The resistance changes from 200 to 250 °C by 2 orders of magnitude, while the resistance by reduction at 300 °C is ∼60% the resistance at 250 °C (Figure 5c).

The temperature range of 200–250 °C is important for reduction, where most of the changes of the GO lattice and functional groups occur.^58^ By 200 °C, drastic vaporization of intercalated H_2_O and a lattice contraction should have taken place, and a partial removal of the main oxygen groups, such as carboxyl and carbonyl, already starts at 200 °C.^59^ This creates graphitic sp^2^ domains enabling charge carrier transport according to the hopping model,^60^ which is also confirmed using XPS and Raman spectroscopy. It results in finite band gaps of the rGO compared to pristine graphene, which is a zero-gap semimetal.^35^ Usually, high temperatures above 1000 °C are required to completely remove residual carboxyl, hydroxyl, and epoxy groups, however, at the cost of increasing disorder levels.^59^ Thus, the reduction temperature has a significant influence and hence affects the rGO properties dramatically. Interestingly, in the range of 200–300 °C, the reduction temperature tuning leads to significant sensing properties of the rGO films. For applications as temperature sensors, the measured temperature range should not exceed 200 °C because any further increase leads to further reduction of the partially reduced GO and causes a declining sensitivity. This limits the operational range of the films, which must be carefully chosen and be significantly below the reduction temperature.

In addition, the electrical resistances of the rGO/PVA composites with different GO:PVA ratios (25%, 50%, 75%, and 90%) reduced at 250 °C are also shown in Figure 5c. The effect of the mixing ratio significantly influences the electrical conductivity below 50%. Above 50%, the effect of the mixing ratio is much lower. The rGO/PVA composite films are less conductive than the rGO films due to the insulating nature of PVA, meaning that PVA limits the electron transport after reduction as it interlaces between the GO layers, as confirmed by SEM and by the excess of oxygen groups observed by XPS.

### Temperature Dependence of the rGO and rGO/PVA Film Resistances

#### Evaluation of the Sensitivity in the Temperature Tested Range 10–80 °C

**Figure 6:**
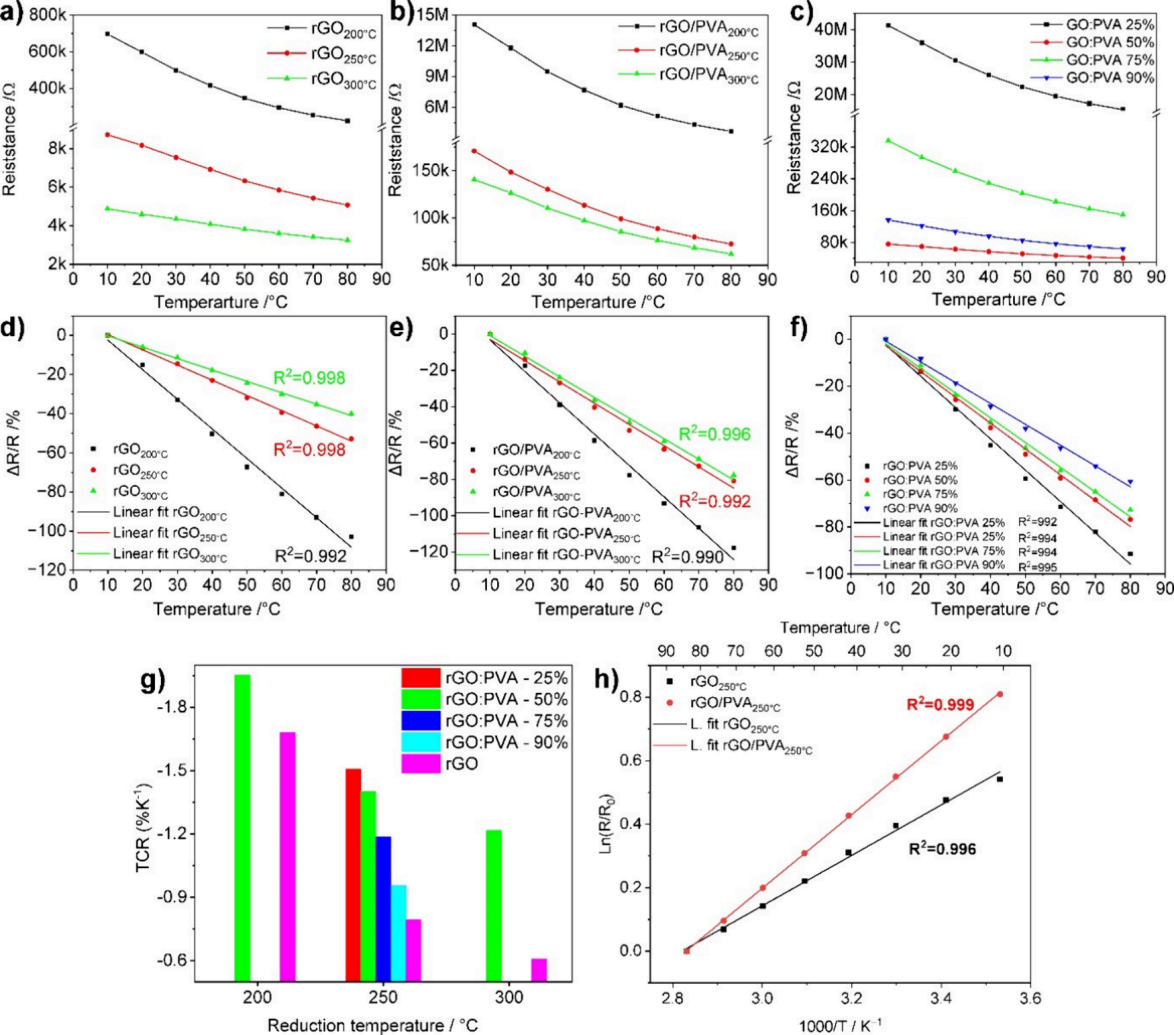
Temperature dependency for (a) rGO and (b) rGO/PVA (50%) reduced at 200, 250, and 300 °C prepared with 50% GO:PVA mixing ratio, (c) rGO/PVA reduced at 250 °C prepared with different GO:PVA mixing ratios of 25%, 50%, 75%, and 90%; (d, e, and f) are the relative changes of resistance versus temperature for (a, b, and c), respectively. (g) TCR for rGO and rGO/PVA at different ratios and reduction temperatures, and (h) logarithm of the relative resistance versus reciprocal temperature for rGO and rGO:PVA 50% reduced at 250 °C.

This section discusses the temperature dependence of the composite film resistances for different mixing ratios and reduction temperatures. The temperature measurements were performed in the range from 10 to 80 °C in a controlled environment where the relative humidity (RH%) was kept at 10%. Figures 6a and 6b show the relation between the resistance of GO and GO/PVA (50 wt %) versus temperature. There is semiconducting behavior, i.e., a negative temperature coefficient (NTC) of both rGO and rGO/PVA films, with the resistance decreasing as the surrounding temperature increases. Figures 6d and 6e show the relative change of the resistance based on eq 1. Moreover, the sensitivity significantly depends on the reduction temperature, whereby increasing the reduction temperature decreases the sensitivity. For rGO/PVA, unlike hydrogels or elastomer polymer-based sensors, where the sensitivity originates from thermal expansion,^39^ the temperature dependence is intrinsically similar to that of rGO films. Thus, the conductivity changes for a semiconductor with a negative temperature coefficient.

It is believed that rGO has a semiconducting behavior as well as a metallic one.^61^ Thus, the charge carriers’ concentration increases by thermal activation upon increasing temperature, leading to a decrease in the resistance. Upon further increase of the temperature, as more charge carriers are generated, the charge scattering increases. Thus, after that point, the resistance decreases at a lower rate. Therefore, the sensitivity declines at high temperatures. At higher reduction temperatures, a higher charge carrier concentration is present due to the removal of oxygen groups and restoration of the sp^2^ hybridization (i.e., the π-electron system). As a result, scattering increases and reduces sensitivity. Thus, scattering plays an essential role in limiting the sensitivity compared to high ohmic resistance films obtained at relatively low temperatures.

Figures 6a and 6b compare the films prepared at different reduction temperatures for rGO and rGO/PVA. The sensitivity of rGO/PVA films reduced at 200, 250, and 300 °C is notably higher than those of rGO produced at the same reduction temperature. The composite-based sensors outperform the rGO ones. This may be attributed to the contribution of the PVA heat capacity and its thermoresponsive properties.^62^ PVA also has a negative temperature coefficient, whereby ascending the temperature, vibrations of the polymer chain release free charges contributing to the conduction process.^43^ In addition, it is suggested that PVA increases the tunneling effect between GO flakes and aggregates, which also increases the activation energy for conduction and, thus, the sensitivity.^63^Figure 6c shows the resistance change for rGO/PVA sensors (different mixing ratios), thermally reduced at 250 °C, and their relative changes in the resistance. In all cases, the resistance decreases as temperature increases. At lower GO/PVA ratios, the sensitivity is higher, which indicates the contribution of PVA for the improvement of the sensing properties compared to rGO films prepared under the same conditions (amount of material and thermal reduction temperature). The improvement of the sensitivity is also supported by the GO percolation in PVA, in addition to the relatively lower concentration of generated mobile charges compared to more conductive films which suffer from scattering, as discussed above. The tunneling effect is more significant in higher-PVA percentage composites, suggesting that the insulating gaps between rGO flakes are larger.^50^

In Figure 6d–f, the relative change of the resistance is presented, calculated according to eq 1:
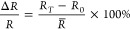
1where *R*_*T*_ and *R*_0_ are the resistance at a given temperature *T* and resistance at the initial temperature, respectively, in Ω, and *R̅* is the average. All curves of the relative change of the resistance were fitted linearly for all cases (*R*^2^ > 0.99). In comparison, rGO-based films show better linearity, which improves with an increase in the reduction of temperature and the GO content. Figure 6g shows the temperature coefficient resistance (TCR in %/K) for rGO and rGO/PVA films reduced at different temperatures and for those with different mixing ratios. It clearly illustrates the effect of the reduction temperature and the composition as discussed above. The results indicate that the highest sensitivity can be obtained at a lower reduction temperature and a lower amount of GO in the composites. Therefore, the sensitivity was significantly improved by mixing GO with PVA. In Figure S7, the change of TCR, pointwise, is shown, which is calculated by eq 2:
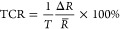
2The TCR is calculated for the entire temperature measurement range and is plotted for all studied films. The sensitivity of the films decreases with increasing ambient temperature, and for highly conductive films, the change of TCR over the temperature range is small. According to the literature,^64^ rGO always shows an NTC behavior, and in the high-temperature range, the conduction is described as an Arrhenius equation with associated activation energy.
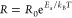
3In eq 3*E*_a_ is the activation energy, *k*_B_ is the Boltzmann constant, *T* is the temperature, and *R* is the resistance. Fitting the resistance–temperature characteristic for the sensors in the high-temperature range (10–80 °C) by a thermal activation Arrhenius-type eq 3 agrees with literature expectations.^65^ The logarithm of *R*/*R*_0_ is a linear function of 1/*T* (see Figure 6h), confirming that the dominant mechanism is band-gap-dependent transport. The calculated activation energies were found 69 and 100 meV for rGO and rGO/PVA, respectively.

#### Evaluation of Temperature Dependence of rGO and rGO and Electron Transport (100–350 K)

**Figure 7:**
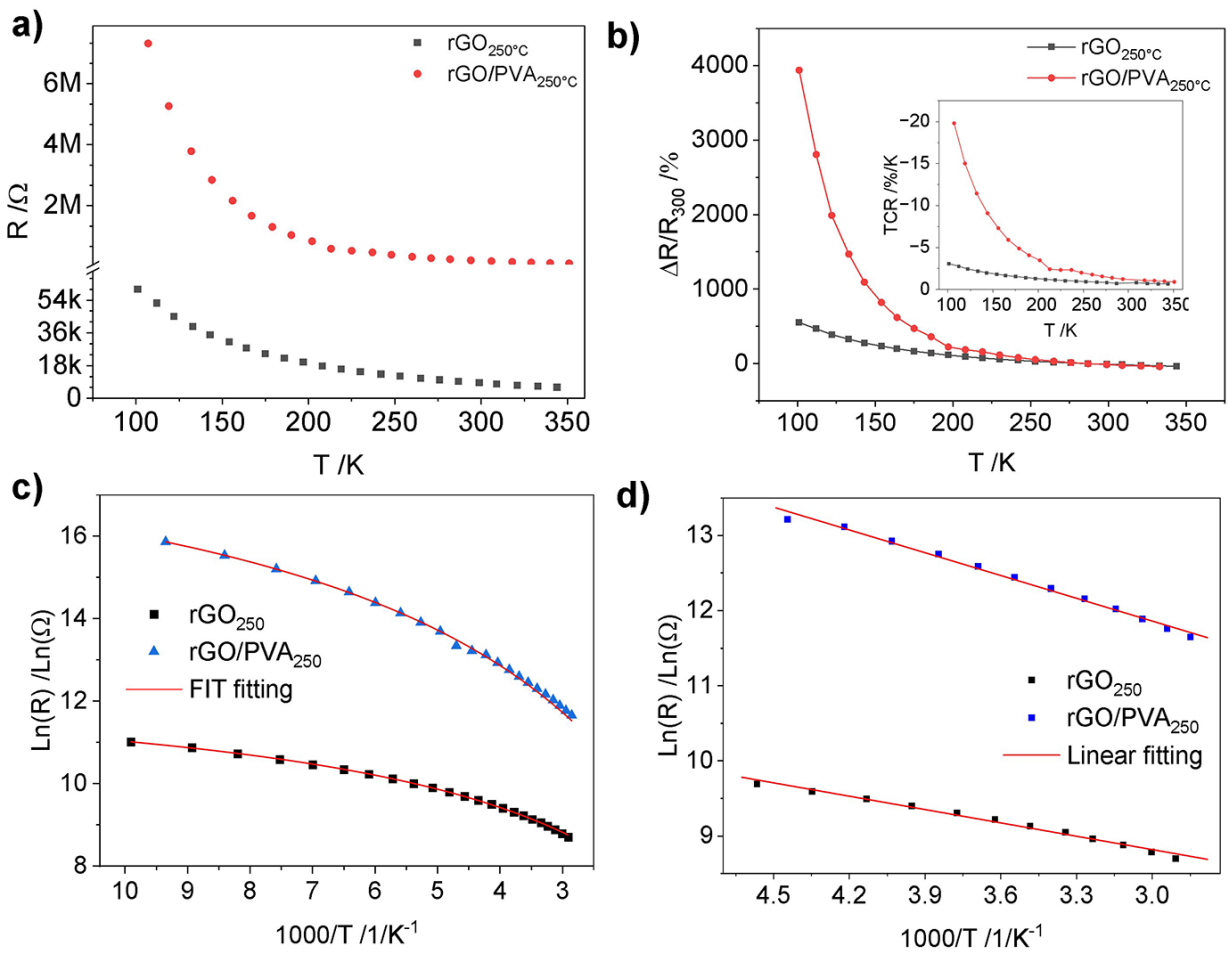
(a) *R*–*T* curve for rGO and rGO/PVA in the temperature range 100–350 K, (b) relative change of the resistance versus temperature; inset is TCR versus temperature, (c) ln(*R*) versus 1/*T* and fluctuation induced tunneling fitting, and (d) linear fit in the range 230–350 K showing an Arrhenius relation.

To further explore the temperature dependency of rGO and rGO/PVA, current–voltage sweeping over a wide range of temperatures from 100 to 350 K was carried out. For both rGO_250 °C_ and rGO/PVA_250 °C_ there is no voltage dependency of resistance since the sensors are ohmic (Figure 7a). The strong dependency of the temperature is pronounced for rGO/PVA where the resistance is decreased (*R*_350 K_/*R*_100 K_) 67 times, or (Δ*R*/*R*_300 K_ ∼ 4000%), in comparison with only 10 times for rGO which indicates the outperformance of rGO/PVA and its possible wide sensing range with higher TCR values in Figure 7b (inset).

To get a better insight into the temperature effects on the transport mechanism, different aspects should be considered, where different transport mechanisms are discussed in the Supporting Information, Section S2.3. As discussed, several mechanisms can be proposed for the transport. By plotting the logarithm of the resistance against the inverse of temperature, fitting the curves (Figure 7c), according to eq S2, represents the FIT model, resulting in good fitting with *R*^2^ = 0.999, in both rGO and rGO/PVA with *T*_1_/*T*_0_ = 8.54 and 14.66 for rGO and rGO/PVA, respectively, which agree with values obtained in ref (66). Linear fitting with good least-squares linearity for both rGO and rGO/PVA films (Figure 7d) in the range 230–350 K suggests an Arrhenius relationship, as discussed in the previous section for the range 10–80 °C.

In the fitting with the hopping VRH mechanism, both Mott and ES-VRH (eq S3) agree only in a small range in the low-temperature part as reported in ref (67), (see Figures S8a and S8b). For the range ∼100−200 K, the reduced activation energy *W* (eq S4) is applied, i.e., plotting ln(*W*) against ln(*T*) and then knowing the slope. The slope obtained is 0.44 for rGO, corresponding to 1/2 2D ES-VRH, and for rGO/PVA it is 0.36, corresponding to 1/3, i.e., Mott-VRH, (see Figures S8c and S8d). This means that there could be a shift from the 2D Coulomb barrier ES-VRH to the 2D Mott-VRH type between rGO and rGO/PVA.

#### Investigation of Sensor Stability and Reproducibility

**Figure 8:**
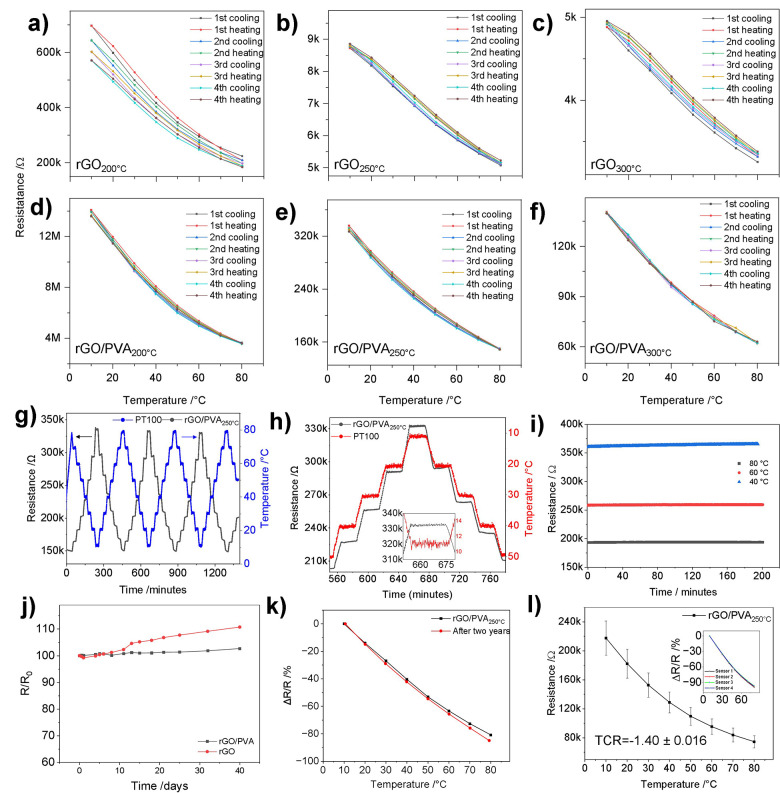
Resistance of rGO and rGO/PVA films versus successive heating and cooling cycles for rGO (a, b, and c) and rGO/PVA (50%) (d, e, and f) at different reduction temperatures, (g) temporal temperature measurement cycles in comparison with PT100 sensor and (h) half a cycle showing the stability of the developed sensor and the inset is a zoom-in to show it stability and small deviation, (i) sensor stability at certain applied temperatures, (j) sensor resistance relative change over time of 40 days, (k) sensor performance comparison within a two year interval, and (l) temperature dependency of rGO/PVA_250 °C_ sensors batch (*n* = 4) of freshly fabricated samples (inset is the relative change).

Figure 8a–f shows the results of repeatability tests, where 4 heating/cooling cycles were carried out for different sensors. For rGO, the repeatability improves at higher reduction temperatures. However, rGO/PVA composites show more stable behavior with almost unchanged resistance and better stability for all reduction temperatures. The hysteresis of the sensors is calculated according to eq 4.^68^
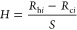
4where *R*_h_ and *R*_c_ are the resistances at the same temperature at *i* heating and cooling cycles and *S* is the sensitivity as *S* = Δ*R*/Δ*T*. The maximum hysteresis was found to be 5.6, 4.5, and 5.6 °C for rGO_200 °C_, rGO_250 °C_, and rGO_300 °C_, respectively. The maximum hysteresis values for rGO/PVA films were 3.2, 2.6, and 2.7 °C for rGO/PVA_200 °C_, rGO/PVA_250 °C_, and rGO/PVA _300 °C_, respectively. The hysteresis drops to less than 0.7 °C for rGO/PVA_200 °C_ and rGO/PVA_250 °C_ in the last cooling/heating cycle compared to 3.3 °C for rGO_200 °C_. Table S1 shows the hysteresis over successive cooling and heating cycles. The presented results indicate improved temperature sensing performance of rGO/PVA compared to the rGO counterpart in terms of sensitivity, stability, and accuracy. The hysteresis even drops in the last cooling/heating cycle, for example, to a maximum of 0.33 °C in rGO/PVA_200 °C_. Figure 8g shows the step temperature change profile for several cycling tests. The developed sensor has repeatable behavior at constant temperature for 30 min with a small deviation of <0.27% (corresponding to 0.2 °C uncertainty), compared to the commercial Pt100 with a temperature deviation of 0.5 °C (see Figure 8g, inset).

Several tests were carried out to prove the stability and repeatability of the sensors based on rGO/PVA_250 °C_. By applying several temperatures for more than 3 h (Figure 8i), the stability factor for resistance variation *C*ν is derived by calculating the ratio of the standard deviation σ and the mean μ of the resistance values over time (*C*ν = σ/μ).^69^ For temperatures of 40, 60, and 80 °C, *Cv* values are 0.39%, 0.09%, and 0.09%, respectively. Figure 8j shows the smaller relative change of the resistance of rGO/PVA_250 °C_ versus time in comparison with rGO_250 °C_, which proves the advantage of rGO/PVA composites. Furthermore, the sensor performance after two years is compared in Figure 8k. It demonstrates sustained sensitivity with slight deviation (maximum 4.08% at 80 °C), indicating that calibration is necessary over time. To prove the reproducibility of the sensor, 4 sensors were fabricated and tested under the same conditions, as shown in Figure 8l. The deviation in resistance values is 10%. However, the sensors have the same relative change within the measured range and measured temperature (Figure 8l), with average TCR = 1.40 ± 0.016%/K.

#### Humidity and Mechanical Dependence of the GO/PVA Films

**Figure 9:**
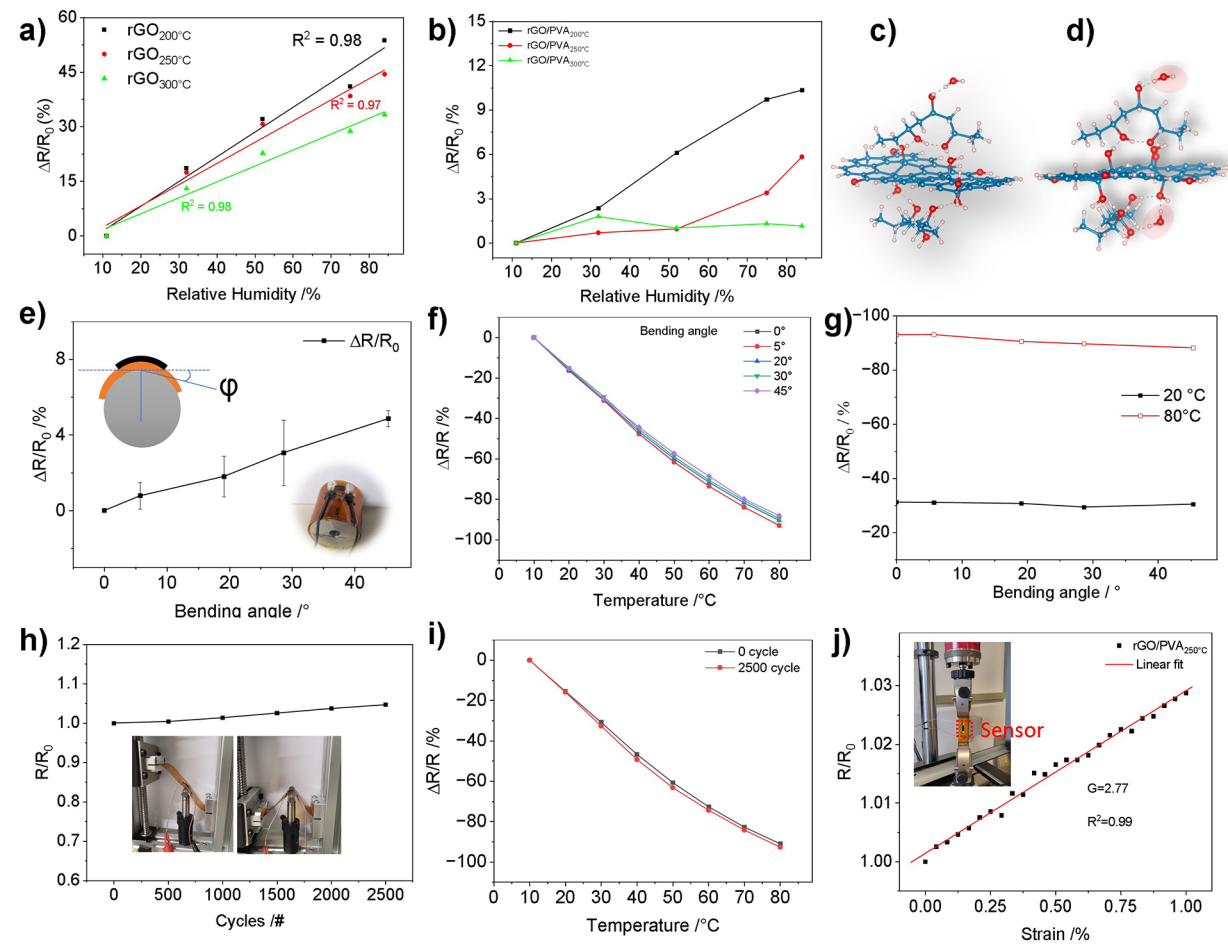
Humidity dependence of (a) rGO and (b) different GO/PVA composite films at different reduction temperatures. (PVA)_2_–rGO model system with (c) one and (d) two water molecules, red ellipses mark H_2_O molecules, (e) relative change of resistance of rGO/PVA250 °C at different bending angles, (f) relative change of the resistance tested under different bending degrees, (g) Δ*R*/*R*_0_ versus temperature dependency for 20 and 80 °C in relation to bending angle, (h) relative change of the resistance versus bending cycle, (i) comparison of sensitivity before and after 2500 bending cycles, and (j) tensile strain sensitivity of rGO/PVA sensor.

The resistance–humidity dependence was analyzed for rGO and rGO/PVA (50%) films reduced at 200, 250, and 300 °C. Figure 9a shows the effect of reduction temperature on RH behavior for rGO. For all rGO films, the resistance increases monotonically with the rise in RH. As described previously, the charge transfer between rGO flakes and water molecules is dominant and causes an increase in impedance.^70^

The relative change of the resistance is presented in Figure 9b for the rGO/PVA composites. Reduction temperatures below 250 °C result in relatively significant sensitivity of the films to RH variation (10–85%), which is due to the remaining oxygen groups on the rGO surface. Still, the maximum relative change is only 10.3% for this reduction temperature. For reduction temperatures of 250 and 300 °C, the effect of RH is minimal (under 3%, Figure 9b). When the humidity is below 40 RH%, a relative change of the resistance is ∼1%, and the change over a wide range of RH% (40–80) is less than 1% for most films. These results indicate that the presented rGO/PVA films are largely humidity-independent temperature sensors. In general, the sensitivity to humidity is 0.04%/RH%, which is much smaller than the average TCR of presented sensors (1.4%/K). The observed insensitivity to humidity is related to the increased hydrophobicity of the rGO surface compared to the starting GO. Water molecules have a negligible influence on the resistance, resulting in a slight increase of the resistance dominated by increased charge transfer and water concentration. At higher humidity, there is no water film formation,^70^ and no proton conductivity was observed for sensors reduced above 200 °C. In addition, the reduction temperature may influence PVA to become waterproof as thermally heated PVA shows only an open circuit (highly ohmic), thus preventing interactions of water with rGO. According to ref (53), heating PVA between 100 and 200 °C can reduce its solubility and water permeability by 500 times, and it can decrease even more by further heating. The influence of humidFity on PVA heated at 300 °C is negligible (see Figure S9) as it is almost insulating. However, at high humidity, the impedance decreases conversely compared to that of rGO. These effects are opposite to each other. Thus, as a result, the rGO/PVA humidity dependence is lowered, which was found by contact angle measurement.

To address the interactions of rGO/PVA with water at the molecular level, we first analyzed the binding of water molecules to the PVA–rGO and (PVA)_2_–rGO models. In the case of the PVA–rGO system, the HOMO–LUMO gap increases slightly with the addition of one water molecule (from 4.172 to 4.214 eV). When one more water molecule is added to the system, the HOMO–LUMO gap increases further, but only by 0.016 to 4.230 eV. The energy released by water adsorption is up to −52 kJ mol^–1^. In the case of the (PVA)_2_–rGO systems, water molecules prefer to interact with the PVA component and not to intercalate between the PVA fragment and the rGO sheet (see Figures 9c and 9d). The water adsorption energy is reduced compared to the system with less PVA, which is up to −39 kJ mol^–1^. Thus, when more PVA is present, the system weakly binds water weaker. Moreover, for the system with a higher PVA amount, the HOMO–LUMO gap is very weakly affected by water adsorption. For the water-free system, it amounts to 4.287 eV, which becomes 4.266 eV after the adsorption of one water molecule and 4.275 eV after binding the second water molecule. A small HOMO–LUMO gap reduction can be linked to the behavior presented in Figure S9 (resistance decrease for nonheated PVA films).

Hence, based on the obtained results and the related discussion, it can be concluded that the reduction of GO/PVA films has a beneficial impact on the humidity response. By inducing the hydrophobic behavior of the rGO/PVA films, no additional calibration or coating by the hydrophobic passivation layer is needed within RH < 80%, as suggested in refs (42 and 43).

The rGO/PVA_250 °C_ sensor batch was examined for several mechanical tests, i.e., temperature measurements for bent sensors at different bending degrees (5°–45°), applying 2500 bending cycles at 45° and strain tensile testing. Figure 9e shows the relative change of the resistance at different bending degrees to have a maximum change of 4% at 45°. The performance of the bent sensors is shown in Figure 9f, and it is a slight decrease in the resistance relative change by increase in the bending degree. In Figure 9g, the Δ*R*/*R*_0_ is shown for 20 and 80 °C. The maximum change is found at maximum bending applied of −4% at 80 °C, demonstrating the sensors’ flexibility and the stability of their sensitivity upon bending application. By applying successive bending cycles of 2500 and measuring the resistance every 500 cycles (Figure 9h), a slight increase in the resistance is found, which is due to the silver paste contact being loose, indicating sensor layer stability. In Figure 9i, temperature sensitivity performance is maintained and is well repeated after bending of 2500 cycles, which proves its durability and flexibility after many bending cycles. A tensile strain test was also applied using a strain machine where up to 1% strain was applied, i.e., 80 N, and an elongation of 1 mm was done for a 12 cm substrate (Figure 9 j). The maximum change of the resistance at 1% is <2.8%. The sensitivity calculated (gauge factor (GF) = Δ*R*/*R*/Δ*l*/*l*) was found to be 2.77. It also indicates a low effect of the strain.

#### Effect of Passivation Coating on the Sensor Performance

**Figure 10:**
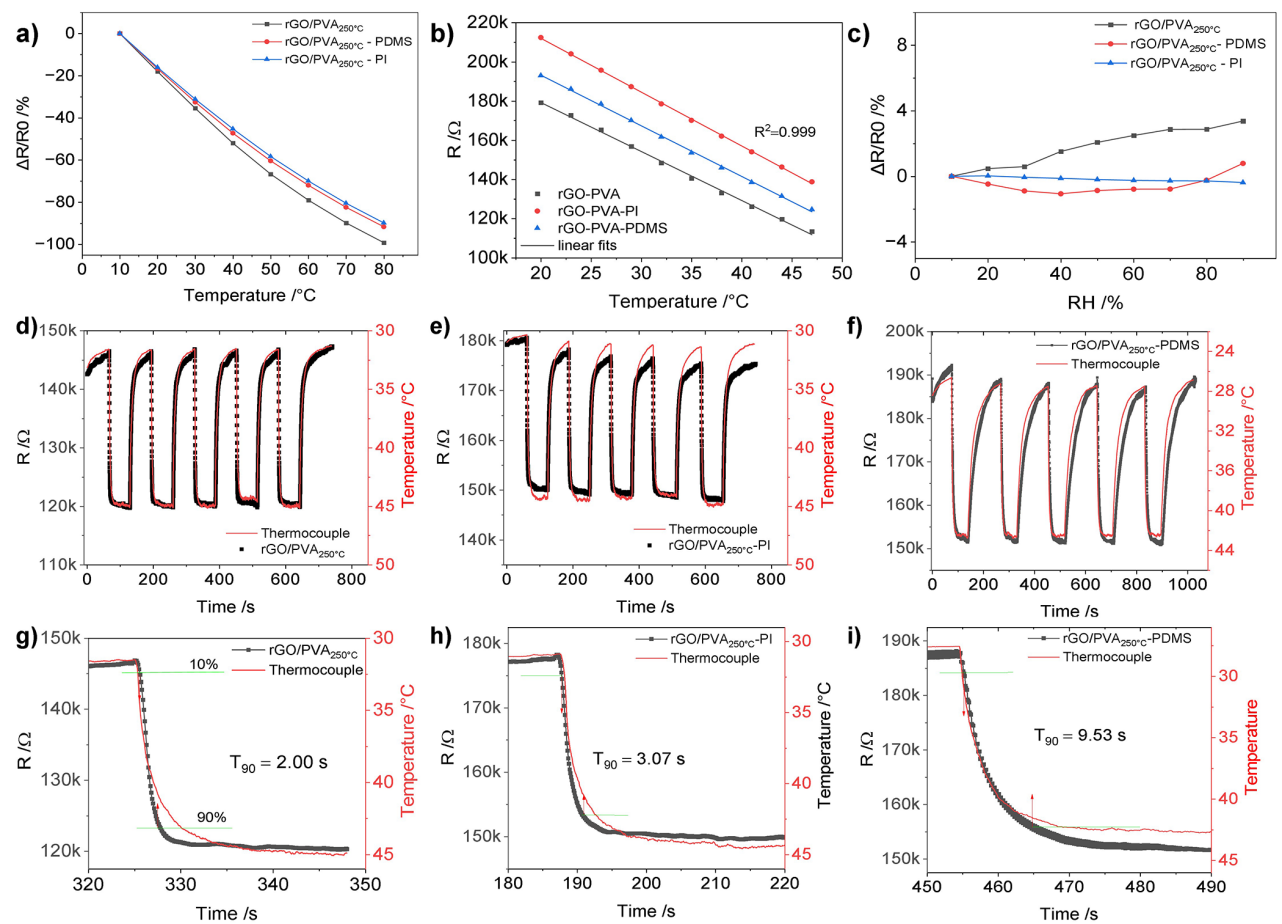
(a) New films based on rGO/PVA_250 °C_ with PI and PDMS coating and their temperature dependency, (b) humidity effect of films in (a) and (c) measured in range the 20–47 °C, (d–f) cycling of the sensor with hot air at 3 m/s, and (g–i) showing the response time for each sensor

In this section, rGO/PVA_250 °C_ sensor sets were fabricated to compare the performance of different coating materials, i.e., PI and PDMS (Figure 10). It was noticed that the coating altered the initial resistance and slightly affected the temperature sensitivity. This set of sensors was tested at the beginning for their temperature sensitivity in Figure 10a where the sensitivity becomes slightly lower in the high-temperature range. This may be attributed to the cohesion between the film and PI tape and PDMS passivation. However, in the low-temperature range (20–47 °C), they perform highly linear where the TCR is calculated to be 1.6%/°C (Figure 10b). With regard to the humidity effect, the set was tested in the range of 10 RH% to 90 RH%, (Figure 10c). It was found that PI coating is efficient in this range with a very small change rate of the resistance (0.005%/RH%), with a maximum change of less than 0.4% at 90%. Meanwhile, the PDMS coating fluctuates, where the change in the middle humidity range at 40 RH% is 1.06%, and at high humidity it is less than 1%. For bare rGO/PVA, it is similar to what was reported in the previous section, where at 90% it is 3.4%. That confirms that a humidity-insensitive sensor can be obtained, especially if the operation range is not highly humid to reach above 80%. The second test was to measure their repeatability under cycling with hot air (hot air, 3 m/s speed, on, hot air off in equal intervals). The results in Figure 10d–f prove the outperformance of rGO/PVA_250 °C_ bare in terms of stability through the cycles. The response times were extracted and averaged for all cycles by averaging the fall times of the resistances for each sensor. The response times (*T*_90_) are 2.13 ± 0.08, 3.38 ± 0.1, and 9.81 ± 0.31 s for rGO/PVA_250 °C_ bare, rGO/PVA_250 °C_-PI and rGO/PVA_250 °C_-PDMS, respectively. The shown *T*_90_ in Figure 10g–i are the smallest values, selected based on certain peaks. The values of the response time indicate that developing an encapsulation-free sensor is very important in applications where response time is critical.

### Investigation under Realistic Conditions and Performance Overview

**Figure 11:**
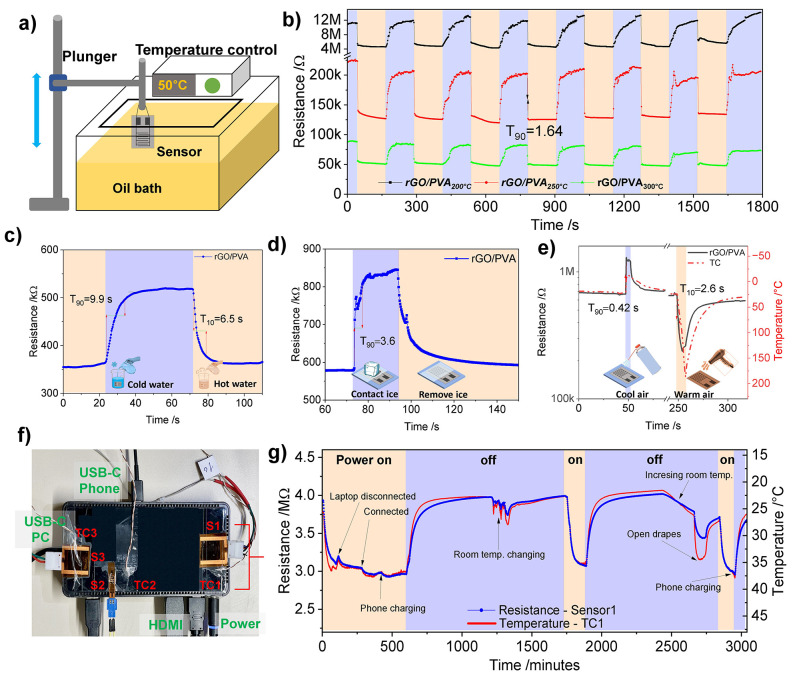
RGO/PVA-based sensors are applied in different simulated environments to apply sudden changes and estimate the response time. (a) Test setup for plunging the sensor in the oil bath, (b) sensors’ results of several cycles by suddenly inserting oil bath. rGO/PVA_250 °C_: (c) response time by application of the sensor on a beaker and filling and removing cold and hot water, (d) response time by applying and removing an ice cube, and (e) response time by blowing fast stream hot/cold air. Real application scenario by monitoring the temperature profile of a docking station (TC = commercial thermocouple) using several sensors (rGO/PVA_250 °C_) in different positions; (f) image of the sensor distribution and (g) results of sensor 1 and TC1 over a long time.

To exploit the sensor properties and applicability, several experiments were carried out to test the reaction of the sensor toward different sudden and/or slow temperature changes (Figures 11, S10, and S11). The first experiment was to insert the sensor suddenly into an oil bath, where the rotating oil was preheated to 50 °C, as shown in Figure 11a. Several sensors were investigated simultaneously, and all display fast changes and repeatable measurements. Furthermore, they prove to withstand heat in the oil medium without any protective coating covering the sensors. The sensor resistance fall time (resistance decrease corresponding to heating) is calculated in most cycles to be 1.64 ± 0.2 s for all sensors, as shown in Figure 11b. In Figures 11c and S10a, inspired by ref (35), the sensor and thermocouple were put on a beaker (bent at 16°). Then, upon filling and emptying the beaker with hot and cold water, repeatable results were obtained, in agreement with the temperature profile obtained by the thermocouple. The rise time (resistance increase corresponding to cooling) and fall time were obtained to be 9.9 and 6.5 s, respectively. In addition, Figures 11d and S10b show how putting an ice cube on the sensor causes a sudden change with a response time of 3.6 s.

Furthermore, the sensor was put on a metallic plate along with K-type thermocouples covered with polymeric tape to prevent any short circuits or icing (Figure S10c). Hot and cold air were applied at different intervals, where a hot air gun and a pressurized cold spray bottle were used. By using cold air, the response time of the sensor was as fast as 0.42 s, and when hot air was used, the response time was 2.6 s (Figure 11e). After several cycles of applying hot and cold pulses, the sensor returns to its initial resistance. This indicates a fast response of the sensor as well as its stability.

Next, the sensitivity and stability of the developed sensors were assessed in a practical measurement scenario of monitoring the temperature change without any mechanical intervention. For this purpose, a setup for monitoring the temperature changes of a laptop docking station was configured with different temperature change scenarios. The developed sensors and K-type thermocouples were attached to the surface of the docking station. The positions of the sensors and the connections are shown in Figure 11f. During the measurement, several actions were carried out to induce different temperature stimuli and to examine if the sensors could follow the changes. All the sensors could follow the temperature profile during the test (2 days with several cycles), as can be seen in Figures 11g and S11.

It can be concluded that GO/PVA-based sensors are reliable and repeatable within single-batch production, from batch-to-batch, and over time so that they can be used for temperature monitoring in different applications such as battery diagnosis, robotics, and wearable devices. As they possess a higher resolution, in the measurement range from 20 to 50 °C (Figures 10c and S7), their application as human wearable temperature sensors is promising. In Table S2, we compare recent literature on temperature sensors based on different nanomaterial films, where the comparison is based on the best figure of merit reported by the papers.

## Conclusion and Outlook

By mixing GO and PVA in different ratios, electrically conductive films have been obtained upon reduction at relatively low temperatures (200–300 °C). The performance of the resulting films was studied for varying GO/PVA mixing ratios and reduction temperatures. In general, the gain in the sensitivity improvement compared to rGO was 1 order of magnitude. The TCR of rGO/PVA_200 °C_ reaches −1.95%/K with good linearity. Based on GO/PVA composites, temperature sensors can be realized, outperforming GO-based sensors alone in terms of stability and hysteresis. The temperature sensitivity is due to the hopping between localized states as well as the fluctuation-induced tunneling gaps between flakes in the low-temperature range while thermal activation dominates the high-temperature range. The investigation results show that PVA contributes to an increase in the tunneling gap and therefore increases the temperature dependence of the resistance. The sensors based on rGO/PVA composite are remarkably humidity insensitive compared to rGO ones. Theoretical calculations suggest that increasing the amount of PVA weakens water–(PVA–rGO) interactions, while the HOMO–LUMO gap is practically unaffected by water adsorption. This agrees with the literature that heat-treated PVA acts as an antiwater coating as heat decreases its water permeability. In addition, mechanical deformation applied on rGO/PVA proved to be stable and only slightly affected its temperature sensing performance. Coating of the sensor by PI or PDMS can improve its humidity independence. However, it can lead to reduced sensitivity and a slower response time. The investigation results show that rGO/PVA composite films are excellent candidates for sensitive, reproducible, repeatable, stable, and humidity-insensitive (<80% RH) temperature sensors with good conformity to be coated on different structures for various applications, including automotive, robotics, wearables, and coating directions such as smart antiviral coatings. As a future prospect, improvement of the sensitivity and response time is aimed at a rather new passivation technique, such as atomic layer deposition to have a waterproof sensor and thus better immunity to ambient air contamination. Since the sensor has proven to have long-term performance, optimization of sensor signal processing is needed to improve the resolution.

## Supporting Information Available

Additional materials, and methods: physical characterization (S1.1) and semiempirical quantum chemistry calculations (S1.2); models of PVA and rGO for semiempirical calculations; Tauc plot for optical band gap determination for rGO and rGO/PVA: Insets: band gap energy versus reduction temperature; AFM image of as-received GO and after sonication; SEM images of GO and rGO/PVA at different mixing ratios; AFM image of GO and rGO deposited films; PVA–rGO model for semiempirical calculations; TCR values for all films; additional information on low-temperature measurements of rGO and rGO/PVA; fitting of temperature dependence by the VRH model; table of cyclic hysteresis of rGO and rGO:PVA(50%); humidity dependency of unreduced GO, PVA, and PVA heated at 300 °C films, testing of the rGO/PVA sensors in different application scenarios; additional information to Figure 11; table comparing different nanocomposite-based temperature sensors (PDF)
