# Supplementary material for: Toward Humidity-Independent Sensitive and Fast Response Temperature Sensors Based on Reduced Graphene Oxide/Poly(vinyl alcohol) Nanocomposites
Source: ACS Appl Electron Mater. 2024 May 31;6(6):4718–34. doi: 10.1021/acsaelm.4c00729 (PMC11210420; doi:10.1021/acsaelm.4c00729)
Supplement: Supplementary file 1 — el4c00729_si_001.pdf [file el4c00729_si_001.pdf]

Supporting Information

Toward Humidity-Independent Sensitive and Fast Response Temperature Sensors Based on Reduced Graphene Oxide/Poly(vinyl alcohol) Nanocomposites

Ammar Al-Hamry[a,*], *Yang Pan*[b], *Mahfujur Rahaman*[b], *Oleksandr Selyshchev*[b], *Christoph Tegenkamp*[c], *Dietrich R. T. Zahn*[b], *Igor A. Pašti*[d] *and Olfa Kanoun*[a,*]

[a]Measurement and Sensor Technology, Chemnitz University of Technology, Reichenhainer Str. 70, 09126, Chemnitz, Germany

[b]Semiconductor Physics, Chemnitz University of Technology, Reichenhainer Str. 70, 09126, Chemnitz, Germany

[c]Analysis of Solid Surfaces, Chemnitz University of Technology, Reichenhainer Str. 70, 09126, Chemnitz, Germany

[d]Faculty of Physical Chemistry, University of Belgrade, Studentski trg 12-16, 11158 Belgrade, Serbia

*Corresponing authors: ammar.al-hamry@etit.tu-chemnitz.de, olfa.kanoun@etit.tu-chemnitz.de

S1 Supporting information to materials and methods

S1.1 Physical characterization

UV-Vis-NIR spectrophotometry was performed on diluted solutions of the various samples using a Cary 60 spectrophotometer (Agilent, USA) by adding 60 µL of the dispersion to 3 mL of distilled water. SEM images of the samples dispersed over silicon substrates were taken using an FEI Nova NanoSEM 200 to examine the quality of the dispersed film morphology and surface properties; all samples here were heated before the experiment. AFM imaging was carried out using a Keysight 5600LS system (Keysight, USA) in tapping mode. The software WSxM was used to process AFM images [1]. The contact angle measurements were carried out with a computer-controlled device (KRÜSS Scientific Instruments, Germany). The determination is performed employing the sessile drop method with deionized water. The Raman spectra were acquired using an excitation wavelength of 532 nm focused with a 100x objective (0.9 N.A.), and laser power of 100 µW measured under the objective. A Horiba Xplora was used with an EMCCD detector and 1200 lines/mm grating providing a spectral resolution of 4 cm^{-1}. The acquisition time was 10 s, accumulated three times for each spectrum.

X-ray photoemission spectroscopy (XPS) experiments were carried out using a Thermo Scientific™ ESCALAB™. 250Xi X-ray Photoelectron Spectrometer. The spectra were acquired with monochromated Al Kα (hv = 1486.68 eV) X-ray source at a pass energy of analyzer of 20 eV, providing a spectral resolution of 0.5 eV. The well-conducting rGO samples were measured without additional compensation or spectra correction, while for acquiring the high-resolution spectra of GO, GO/PVA, and rGO/PVA samples, a flood gun with low kinetic energy electrons was applied to prevent the charging of the sample. The Avantage Data System (Thermo Scientific™) was used to quantify and deconvolve the spectra.

S1.2 Semi-empirical quantum chemistry calculations

Semi-empirical calculations were performed using the MOPAC2016 code [2] with the PM7 method [3], and the MoCalc2012 code as the user interface [4]. Full structural relaxation was performed. HOMO-LUMO gaps were determined for different PVA-rGO model architectures. Visualization was done using Jmol [5] and VESTA [6]. The models were made by combining the rGO model cluster with PVA formed of three monomeric units. The rGO cluster had the formal stoichiometry of $C_{56}H_{32}O_8$, thus having a C:O ratio of 7. The model contained four phenolic C-OH groups located at the edge sites and four hydroxyl groups at the basal plane, grouped into two dimers so that two OH groups are above and two below the rGO basal plane [7]. The models of PVA and rGO are shown in Figure S1 (see supplementary information). The HOMO-LUMO gap of PVA was above 13 eV, while the HOMO-LUMO gap of the rGO models was found to be 4.23 eV. Please note that here we do not aim to reproduce experimental values but to explain experimentally observed trends.

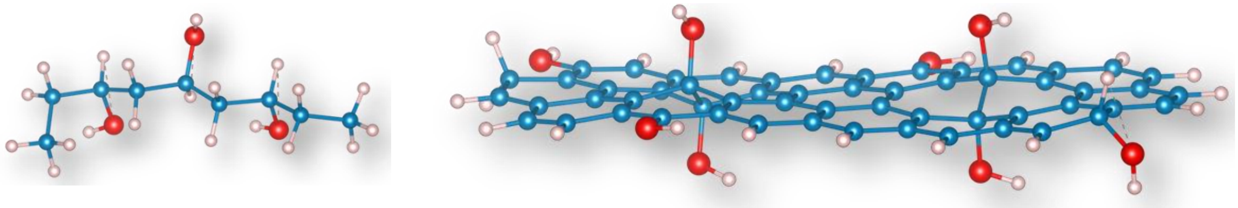

Figure S1. Models of PVA (left) and rGO (right)

S2 Supplementary to Section 3 Results and Discussion

S2.1 Physical characterization

Figure S2 presents the Tauc plots for GO and GO/PVA based films which were plotted according to the following equation S[8]

$$\alpha h\nu = B(h\nu - E_g)^{1/2} \tag{S1}$$

where α is the absorption coefficient, B is a constant, hv is the photon energy, and 1/2 is considered for direct allowed transitions. The band gap was determined by fitting the spectra's linear region and taking the linear fit's intercept with the x-axis. Insets in both figures show the energy band gap versus the reduction temperature.

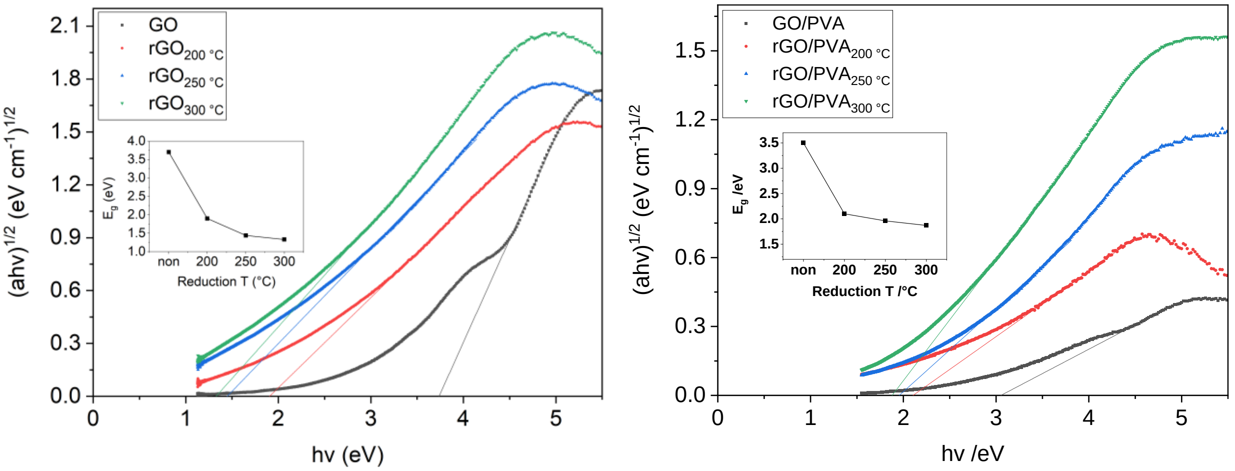

Figure S2. Tauc plot for optical band gap determination for a) rGO and b) rGO/PVA, Insets: band gap energy versus reduction temperature

Figure S3a shows the AFM tapping mode image of the received GO, which was diluted 20 times and drop casted on a silicon substrate. Individual flakes are seen in different positions and measured, indicated by the line on the image, where z-axis roughness is attached in Figure S3c. In Figure 3Sb, GO was first diluted to 0.2% and sonicated at 15% of total power by tip sonication for 30 minutes. To be able to take the images, it was further diluted 20 times. In this image, we see the flakes lying over the substrate with atomic thickness, as can be noticed in Figure 3d. Some agglomeration of smaller flakes for which the highest roughness is attributed can be found. In Figure S4, no dilution after sonication. Spin-coated samples show a highly smooth surface of both GO and GO/PVA. However, it is difficult to distinguish 2D graphene features due to the densely coated tiny flakes, and roughness values are very small.

Figures S5 depicts several images at various mixing ratios. The swelling and breaking of deposited films are evident at high GO percentages, i.e., over 75%, owing to the annealing method employed to reduce GO. It was observed that the 50% mixing ratio gives both structural performance and good electrical conductivity while maintaining good sensitivity compared to other ratios.

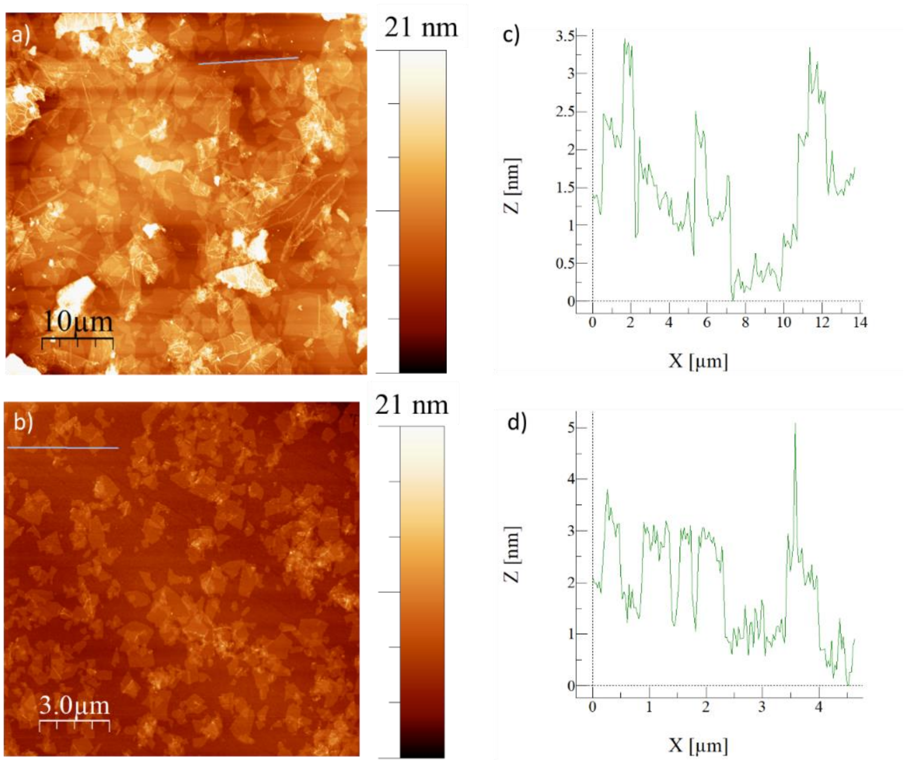

Figure S3 a) AFM image of GO as received and b) after sonication of 30% power for 30 minutes, profile of the marked lines are shown in c) and d).

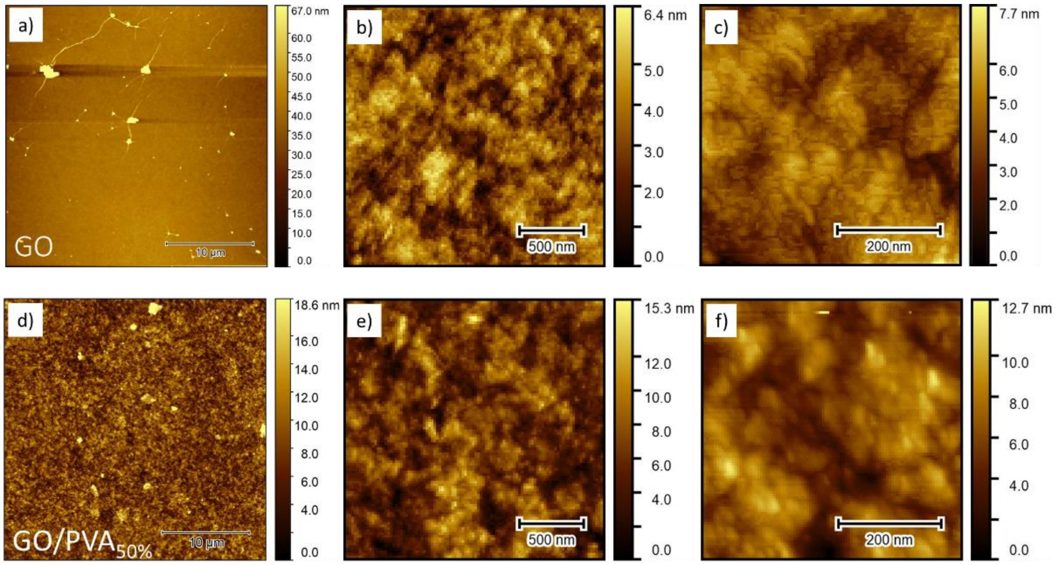

Figure S4 AFM images of rGO at different image sizes (a: 30 µm, b: 2 µm, and c: 500 nm) and rGO/PVA at different image sizes (d: 30 µm, e: 2 µm, and f: 500 nm)

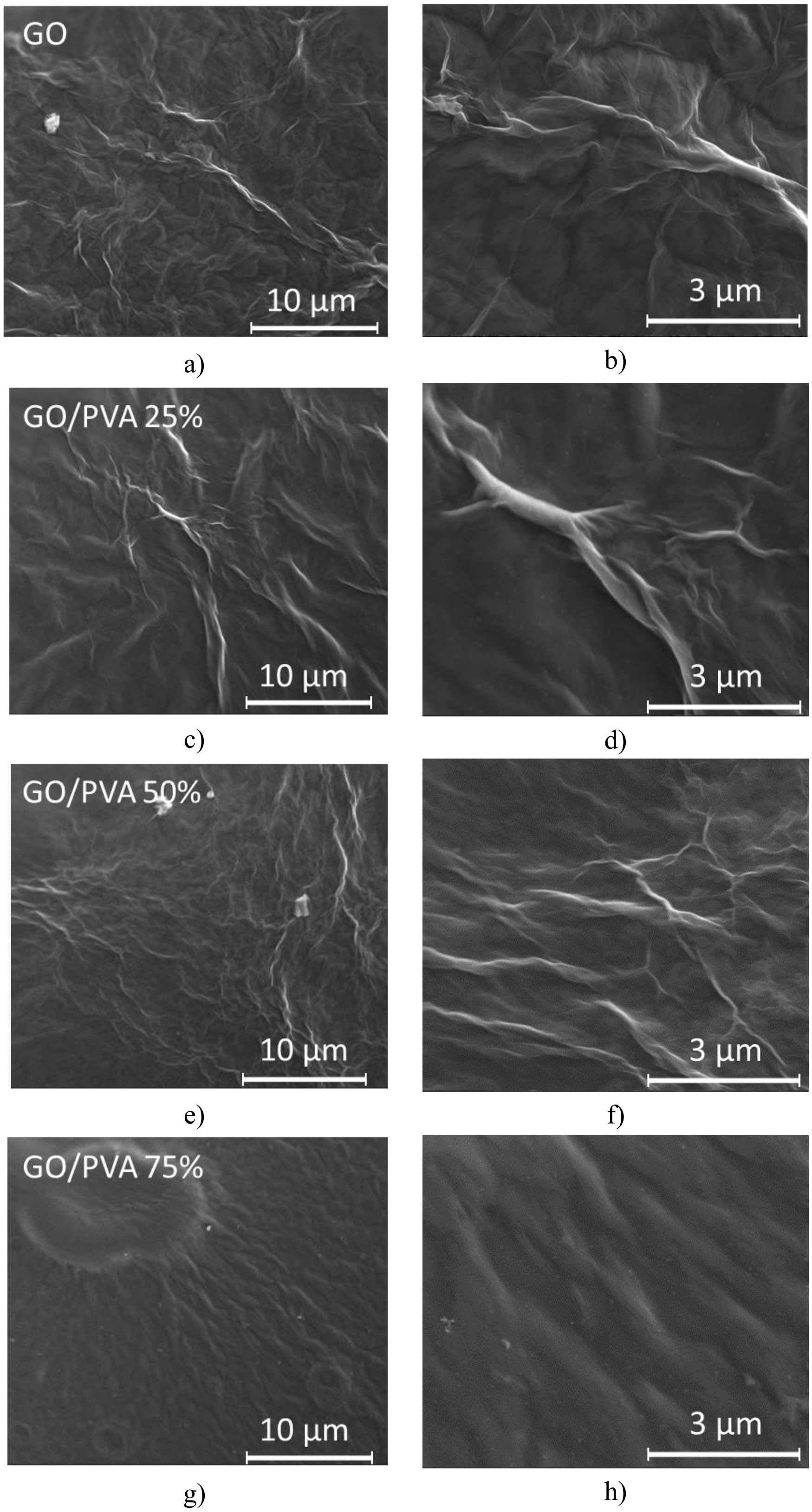
GO
10 µm
3 µm
a)
b)
GO/PVA 25%
10 µm
3 µm
c)
d)
GO/PVA 50%
10 µm
3 µm
e)
f)
GO/PVA 75%
10 µm
3 µm
g)
h)

Figure S5 SEM images for different GO:PVA ratios where all are reduced samples of a-b) reduced GO, c-d) rGO/PVA 25%, e-f) rGO/PVA 50%, and g-h) rGO/PVA 75%

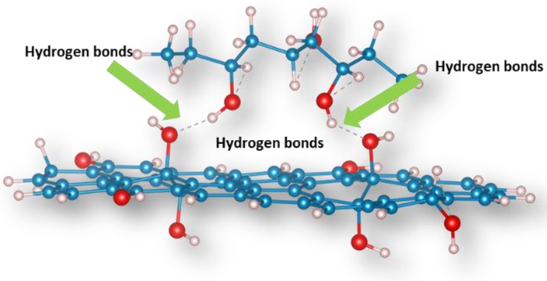

(a)

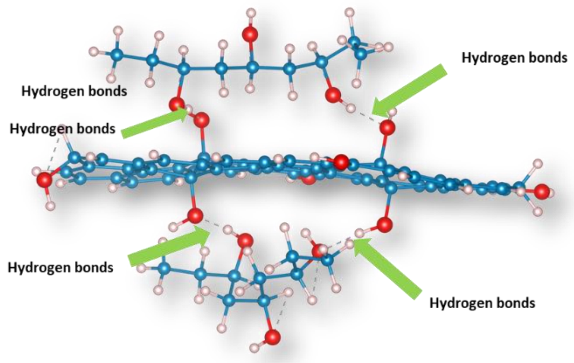

(b)

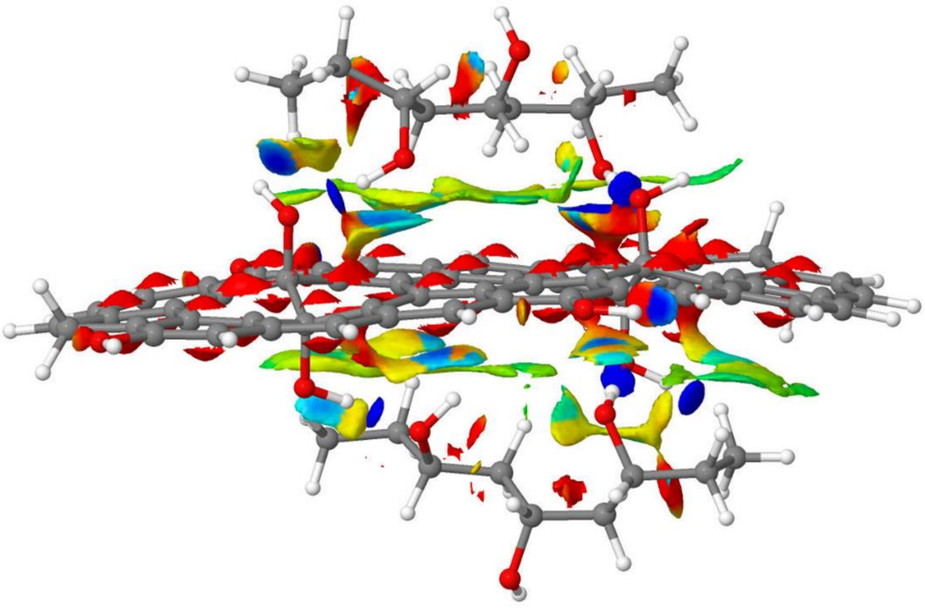

(c)

Figure S6. (a) the PVA-rGO model with indicated hydrogen bonds between PVA and rGO, (b) the (PVA)2-rGO model with indicated hydrogen bonds between PVA and rGO, (c) the (PVA)2-rGO model with depicted regions of non-covalent interactions.

S2.2 Temperature measurement

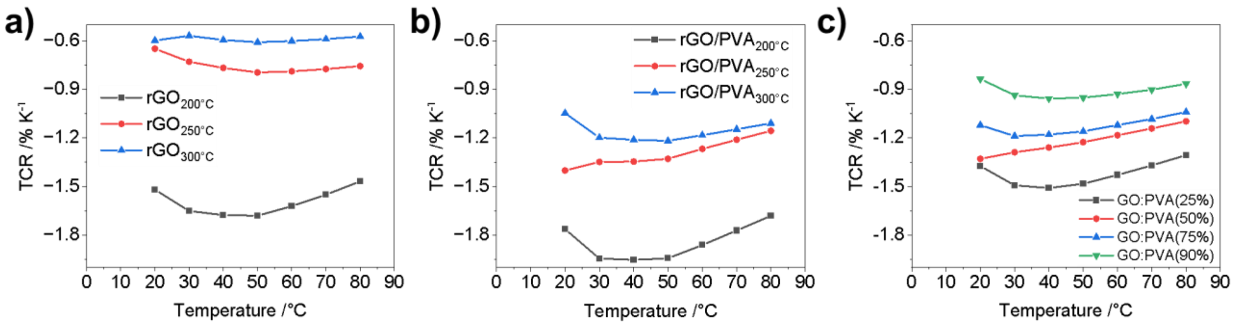

Figure S7 TCR change versus temperature for a) rGO sensors reduced at different temperatures, and b) rGO/PVA (50%) reduced at different temperatures and c) rGO/PVA with different mixing ratios reduced at 300 °C

S2.3 Temperature dependence (100 – 350 K)

For chemically reduced GO/PVA[9], fluctuation-induced tunneling (FIT) was reported as a possible mechanism, as shown in equation S2 [10].

$$\mathrm{R} = R_0 \exp\left(\frac{T_0 + T}{T_1}\right) \quad \text{(S2)}$$

T_1 represents the energy required to overcome the energy barrier, and T_0 is the temperature below which elastic tunneling conduction is the main mechanism [11]. In fluctuation-induced tunneling (FIT) mechanism, charges tunnel from one to another reduced graphene flake through the insulating barrier made of PVA. As the amount of graphene sheets to PVA increases, the tunneling gap becomes smaller. Going closer to room temperatures, transport via thermally activated carriers becomes the dominant mechanism.

In addition, the transport properties of rGO as a function of progressive reduction treatment were reported in [12]. Variable range hopping (VRH) was considered the responsible mechanism for lightly reduced GO, whereas band transport was suggested for highly reduced rGO. A high GO reduction level increases the localized states, which can be expressed at a low-temperature regime just as VRH transport mechanism as in equation S3 [13].

$$R(T) = R_0 \exp\left[\left(\frac{T_0}{T}\right)^m\right] \quad \text{(S2)}$$

R_0 is constant, T_0 is the Mott characteristic temperature, and m=1/3 and 1/4 for 2D- and 3D-Mott-VRH, respectively, and m=1/2 for Efros−Shklovskii (ES)-VRH, which considers the Coulomb interaction. In order to distinguish the type of VRH, reduced activation energy W is plotted against ln(T) (as inn equation S4) [14].

$$W = -\frac{d ln\rho(T)}{d ln(T)} \quad \text{(S4)}$$

According to some sources, all these mechanisms could contribute simultaneously to the transport, especially in large area rGO films [15]. For thermally reduced GO mixed with PVA as composite films, the carriers hopping between the edge-localized states at the rGO-amorphous PVA interface was reported as the transport mechanism at low temperatures [16]. Thermal activation was, on the other hand, regarded as the conduction mechanism in the high-temperature range around room temperature.

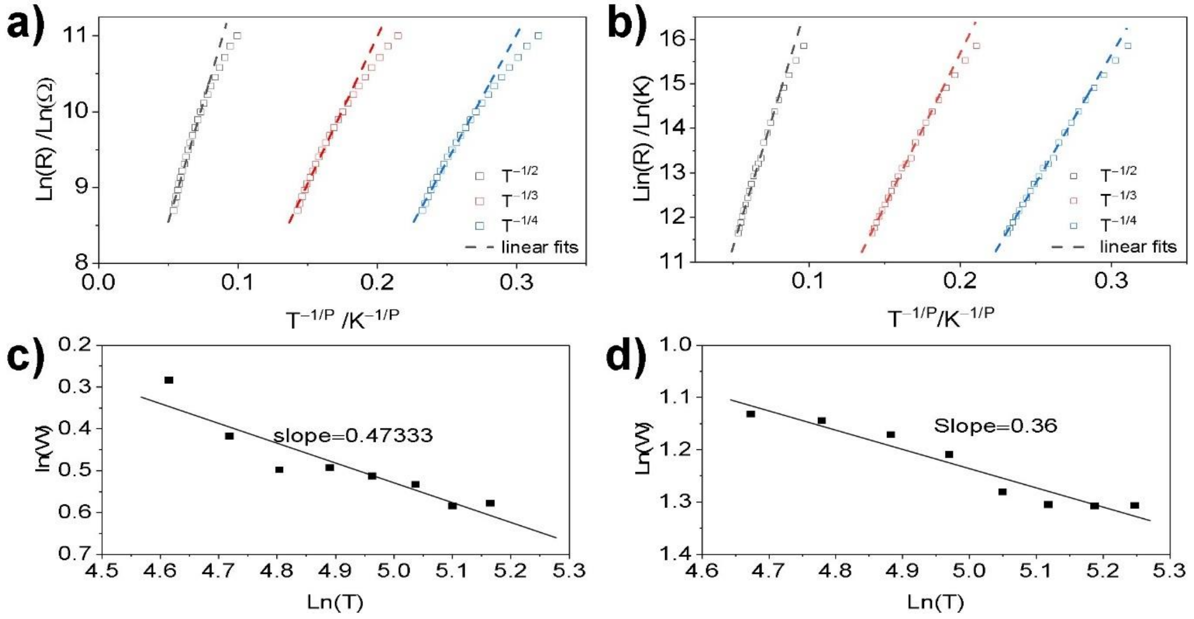

Figure S8 Fitting of rGO (a) and rGO/PVA (b) with VRH model with p=1/2, 1/3, ¼ in the range 100 – 200K and c) and d) are the fitting of ln(W) versus ln(T) for rGO and rGO/PVA, respectively.

Table S1 Calculated hysteresis (°C) of rGO and rGO:PVA(50%) sensors

Cycle number	$rGO_{200°C}$	$rGO_{250°C}$	$rGO_{300°C}$	$rGO/PVA_{200°C}$	$rGO/PVA_{250°C}$	$rGO/PVA_{300°C}$
2: heating	4.97	4.58	5.68	3.19	2.37	2.37
3: cooling	5.01	0.74	2.71	0.92	0.81	0.64
4: heating	5.66	0.74	1.49	1.17	0.81	1.59
5: cooing	4.15	0.71	1.27	0.99	0.66	1.61
6: heating	4.29	0.79	0.83	0.99	0.77	2.43
7: cooling	3.33	1.51	1.04	0.62	0.59	1.64
8: heating	3.33	1.63	1.32	0.67	0.59	2.69

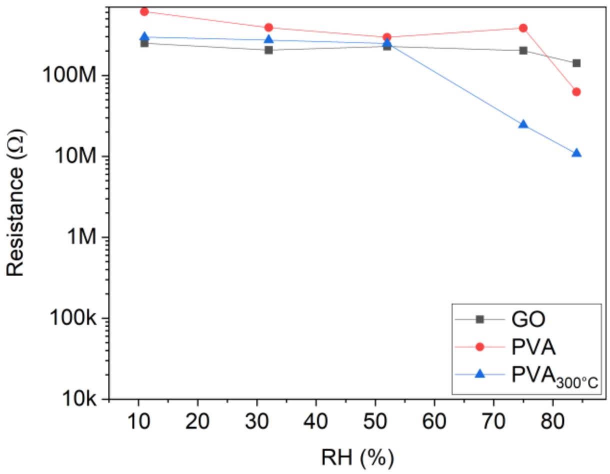

Figure S9 Humidity dependency of unreduced GO, PVA and PVA heated at 300 °C films

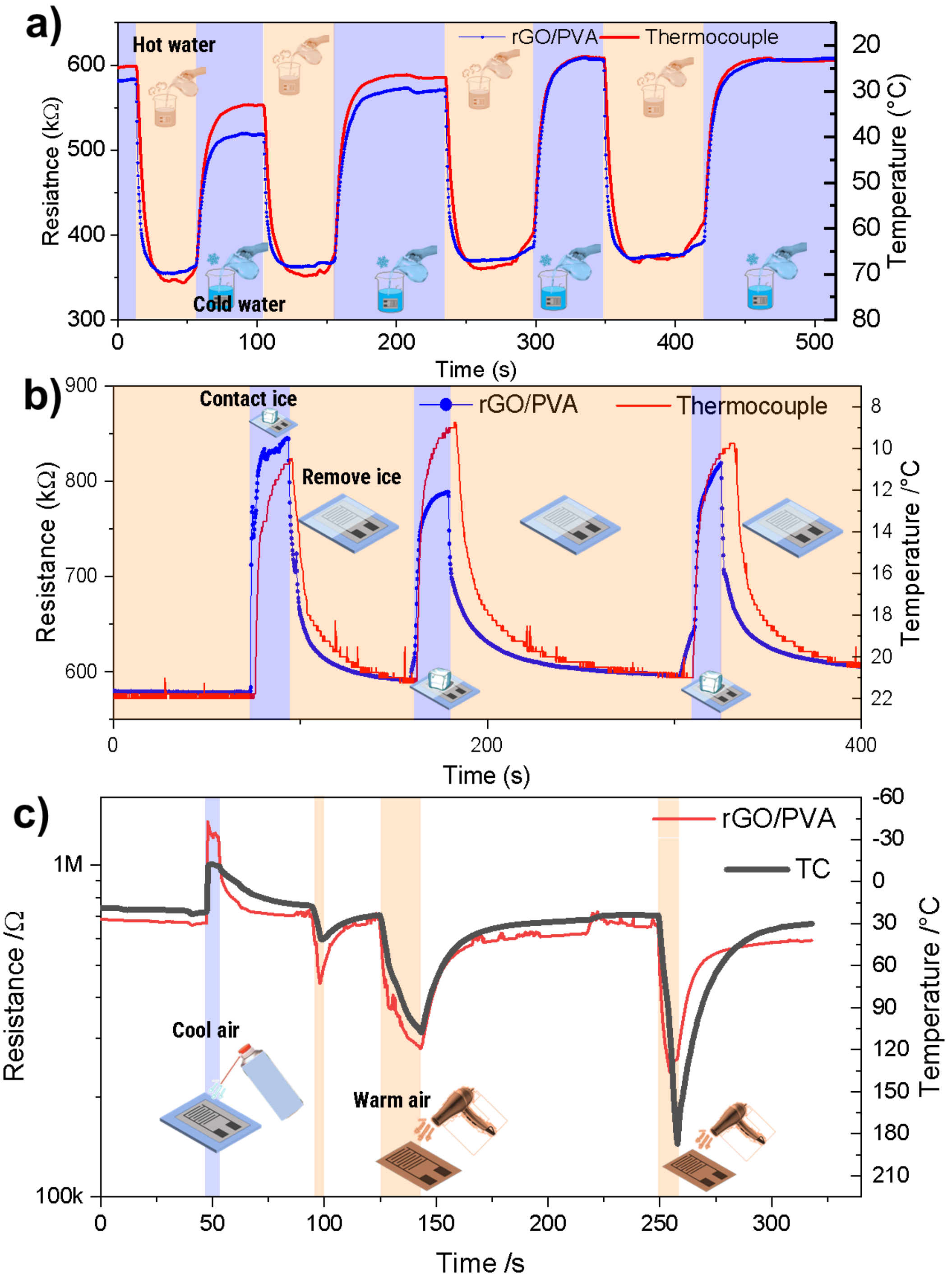

Figure S10 a) Cycles of hot-cold water application on a beaker where the sensor is attached to the outer wall, b) cyclic measurement by putting ice on top of covered sensors, and c) application of hot and cold air on the sensor

Developed sensors along with commercial K-type thermocouples were attached to the surface of the docking station, which has different ports, e.g., USB-C to the laptop, another USB-C to charge a mobile device, and 2 HDMI connected to computer monitors, USBs to keyboard and the power port to the charger. Two large sensors (1.5×2 cm^2), small IDE sensors (0.5× 0.5 cm2), and three thermocouple sensors were used. It can be seen that the sensors follow the temperature profile created over 48 hours. The temperature change is in accordance with the event created by the operator or by room temperature, as indicated in Figure S10. The following can be concluded:

- Temperature changes following power consumption can be seen.
- Thin film polymeric substrate-based sensors have better sensitivity to the temperature.
- They also have better conformity as they can be well attached to the body of the docking device ad have better contact with the body.
- Thermocouples underestimate the temperature by 5 °C, as confirmed by measuring the temperature at different positions by an infrared temperature sensor. That makes the developed sensors high-performance.
- A rise in temperature due to sun irradiation was detected while the power was off.
- Minor changes of power created by adding and removing devices were detected successfully.

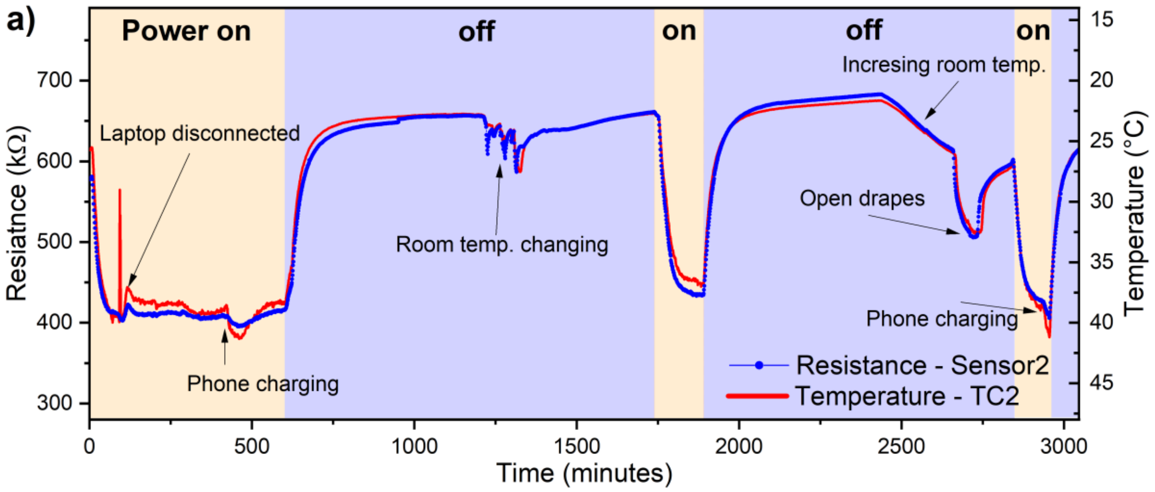

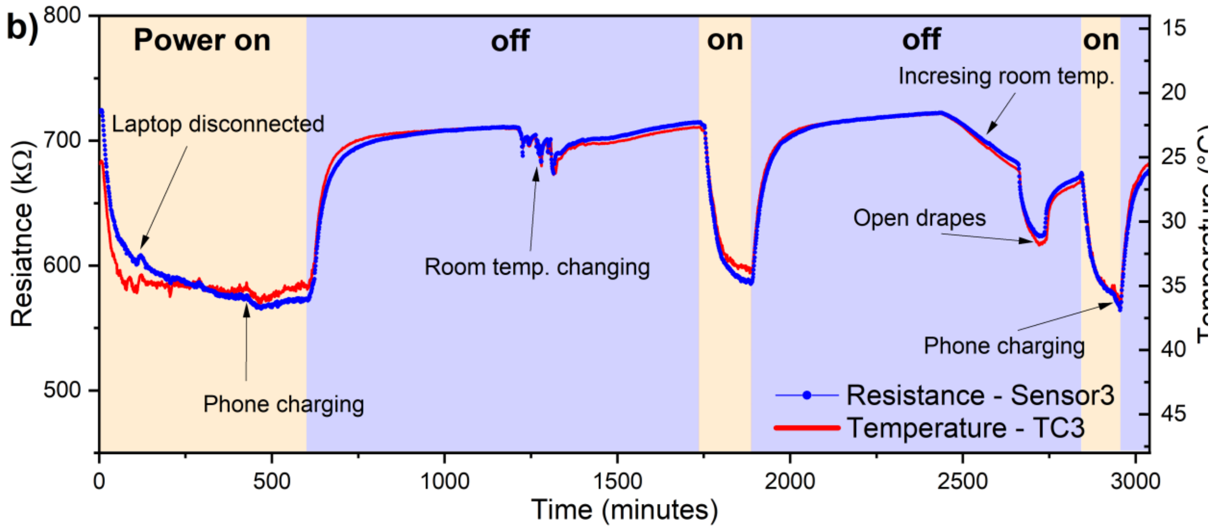

Figure S11 The results of a) sensor 2 and b) sensor 3 positioned in the docking station as shown in Figure 11d

Table S2. Comparison of nanocomposite-based temperature sensor

Material	TCR (%·C°$^{-1}$)	Temperature range (°C)	Response/recovery time (s)	Hysteresis	Ref.
rGO	−0.63	30-100	1.2	-	[17]
rGO-Ag nanocomposite	−0.16	−60-170	0.47	1%	[18]
graphene/PEDOT:PSS×	−0.06	35-45	20/18	RH dependence	[19]
LrGO×	−0.37	30-100	0.19/9.7		[20]
rGO/PU×	−0.36	20-90	-	-	[21]
CNTSs/CB in PVA/Gly hydrogel×	−0.93	30-90	-	-	[22]
G/P(VDF-TrFE)×	+0.61	−20-300	4/3	-	[23]
AgNPs × printed ink	+0.17	20-80	-	0.28%	[24]
PAN/GO fiber×	−0.4	20-100	-	Obvious	[25]
PANI/rGO film×	−0.76	10-80	1.43/15		[26]
Ag, Cu, and Ni nanoparticles ink	0.1-0.23	22-150	-	-	[27]
MWCNT-Ag-PVDF×	−0.18		11	-	[28]
LIS/LIG	− 0.58	30-90	-	-	[29]
rGO/CS	−1.379	30–60	2.4/3.9	-	[30]
rGO/PVA50%-200°C<br>rGO/PVA50%-250°C<br>rGO/PVA50%-300°C	− 1.8<br>− 1.4<br>− 1.22	10-80	0.4/2.6§	0.67 °C*<br>0.59 °C*<br>2.69 °C*	This work

×G: Graphene, LrGO: laser reduced GO, PU: Polyurethane, CNTSs/CB/PVA/Gly: carbon nanotubes/carbon black/poly(vinyl alcohol)/glycerol, P(VDF-TrFE): Poly(vinylidene fluoride-co-trifluoroethylene), Fluoropolymer resin, AgNPs: silver nanoparticles, PEDOT:PSS: poly(3,4-ethylenedioxythiophene) polystyrene sulfonate, PAN: polyacrylonitrile, rGO/CS: rGO/chitosan.
§ Response time given for $rO/PVA_{250°C}$, * Hysteresis at 4th heating/cooling cycle

S3 References

(1) Horcas, I.; Fernández, R.; Gómez-Rodríguez, J. M.; Colchero, J.; Gómez-Herrero, J.; Baro, A. M. WSXM: a software for scanning probe microscopy and a tool for nanotechnology. *The Review of scientific instruments* **2007**, *78* (1), 13705. DOI: 10.1063/1.2432410.

(2) [J.J.P. Stewart. *Stewart computational chemistry - MOPAC*. http://openmopac.net/ (accessed 2022-11-28).

(3) Stewart, J. J. P. Optimization of parameters for semiempirical methods VI: more modifications to the NDDO approximations and re-optimization of parameters. *Journal of molecular modeling* **2013**, *19* (1), 1–32. DOI: 10.1007/s00894-012-1667-x. Published Online: Nov. 28, 2012.

(4) Depizzol, D. B.; Paiva, M. H. M.; Dos Santos, T. O.; Gaudio, A. C. MoCalc: a new graphical user interface for molecular calculations. *Journal of computational chemistry* **2005**, *26* (2), 142–144. DOI: 10.1002/jcc.20151.

(5) Jmol: an open-source Java viewer for chemical structures in 3D.

(6) Momma, K.; Izumi, F. VESTA 3 for three-dimensional visualization of crystal, volumetric and morphology data. *J Appl Crystallogr* **2011**, *44* (6), 1272–1276. DOI: 10.1107/S0021889811038970.

(7) Dobrota, A. S.; Gutić, S.; Kalijadis, A.; Baljozović, M.; Mentus, S. V.; Skorodumova, N. V.; Pašti, I. A. Stabilization of alkali metal ions interaction with OH-functionalized graphene via clustering of OH groups – implications in charge storage applications. *RSC Adv.* **2016**, *6* (63), 57910–57919. DOI: 10.1039/C6RA13509A.

(8) Tauc, J. Optical properties and electronic structure of amorphous Ge and Si. *Materials Research Bulletin* **1968**, *3* (1), 37–46. DOI: 10.1016/0025-5408(68)90023-8.

(9) Mitra, S.; Banerjee, S.; Chakravorty, D. Tunneling conduction in graphene/(poly)vinyl alcohol composite. *Journal of Applied Physics* **2013**, *113* (15), 154314. DOI: 10.1063/1.4802664.

(10) Voit, J.; Büttner, H. Fluctuation-induced tunneling and the conduction mechanism in metallic polyacetylene. *Solid State Communications* **1988**, *67* (12), 1233–1237. DOI: 10.1016/0038-1098(88)91090-3.

(11) Xie, H.; Sheng, P. Fluctuation-induced tunneling conduction through nanoconstrictions. *Phys. Rev. B* **2009**, *79* (16). DOI: 10.1103/PhysRevB.79.165419.

(12) Eda, G.; Mattevi, C.; Yamaguchi, H.; Kim, H.; Chhowalla, M. Insulator to Semimetal Transition in Graphene Oxide. *J. Phys. Chem. C* **2009**, *113* (35), 15768–15771. DOI: 10.1021/jp9051402.

(13) Mott, N. F.; Davis, E. A. *Electronic processes in non-crystalline materials*, 2nd ed.; International series of monographs on physics; Clarendon Press, 2012.

(14) Joung, D.; Khondaker, S. I. Efros-Shklovskii variable-range hopping in reduced graphene oxide sheets of varying carbon sp2 fraction. *Phys. Rev. B* **2012**, *86* (23). DOI: 10.1103/PhysRevB.86.235423.

(15) Haque, A.; Mamun, M. A.-A.; Taufique, M. F. N.; Karnati, P.; Ghosh, K. Temperature Dependent Electrical Transport Properties of High Carrier Mobility Reduced Graphene Oxide Thin Film Devices. *IEEE Trans. Semicond. Manufact.* **2018**, *31* (4), 535–544. DOI: 10.1109/TSM.2018.2873202.
(16) Mitra, S.; Mondal, O.; Saha, D. R.; Datta, A.; Banerjee, S.; Chakravorty, D. Magnetodielectric Effect in Graphene-PVA Nanocomposites. *J. Phys. Chem. C* **2011**, *115* (29), 14285–14289. DOI: 10.1021/jp203724f.
(17) Liu, G.; Tan, Q.; Kou, H.; Zhang, L.; Wang, J.; Lv, W.; Dong, H.; Xiong, J. A Flexible Temperature Sensor Based on Reduced Graphene Oxide for Robot Skin Used in Internet of Things. *Sensors (Basel, Switzerland)* **2018**, *18* (5). DOI: 10.3390/s18051400. Published Online: Feb. 5, 2018.
(18) Neella, N.; Gaddam, V.; M.M., N.; N.S., D.; K., R. Scalable fabrication of highly sensitive flexible temperature sensors based on silver nanoparticles coated reduced graphene oxide nanocomposite thin films. *Sensors and Actuators A: Physical* **2017**, *268*, 173–182. DOI: 10.1016/j.sna.2017.11.011.
(19) Vuorinen, T.; Niittynen, J.; Kankkunen, T.; Kraft, T. M.; Mäntysalo, M. Inkjet-Printed Graphene/PEDOT:PSS Temperature Sensors on a Skin-Conformable Polyurethane Substrate. *Scientific reports* **2016**, *6*, 35289. DOI: 10.1038/srep35289. Published Online: Oct. 18, 2016.
(20) Chen, R.; Luo, T.; Da Geng; Shen, Z.; Zhou, W. Facile fabrication of a fast-response flexible temperature sensor via laser reduced graphene oxide for contactless human-machine interface. *Carbon* **2022**, *187*, 35–46. DOI: 10.1016/j.carbon.2021.10.064.
(21) Xiao, W.; Wang, L.; Li, B.; Li, Y.; Wang, Y.; Luo, J.; Huang, X.; an Xie; Gao, J. Interface-engineered reduced graphene oxide assembly on nanofiber surface for high performance strain and temperature sensing. *Journal of Colloid and Interface Science* **2022**, *608* (Pt 1), 931–941. DOI: 10.1016/j.jcis.2021.10.032. Published Online: Oct. 9, 2021.
(22) Gu, J.; Huang, J.; Chen, G.; Hou, L.; Zhang, J.; Zhang, X.; Yang, X.; Guan, L.; Jiang, X.; Liu, H. Multifunctional Poly(vinyl alcohol) Nanocomposite Organohydrogel for Flexible Strain and Temperature Sensor. *ACS applied materials & interfaces* **2020**, *12* (36), 40815–40827. DOI: 10.1021/acsami.0c12176. Published Online: Aug. 27, 2020.
(23) Mahmoud, W. E.; Al-Bluwi, S. A. Development of highly sensitive temperature sensor made of graphene monolayers doped P(VDF-TrFE) nanocomposites. *Sensors and Actuators A: Physical* **2020**, *312*, 112101. DOI: 10.1016/j.sna.2020.112101.
(24) Zikulnig, J.; Hirschl, C.; Rauter, L.; Krivec, M.; Lammer, H.; Riemelmoser, F.; Roshanghias, A. Inkjet printing and characterisation of a resistive temperature sensor on paper substrate. *Flex. Print. Electron.* **2019**, *4* (1), 15008. DOI: 10.1088/2058-8585/ab0cea.
(25) Ke, F.; Song, F.; Zhang, H.; Xu, J.; Wang, H.; Chen, Y. Layer-by-layer assembly for all-graphene coated conductive fibers toward superior temperature sensitivity and humidity independence. *Composites Part B: Engineering* **2020**, *200*, 108253. DOI: 10.1016/j.compositesb.2020.108253.
(26) Al-Hamry, A.; Lu, T.; Bai, J.; Adiraju, A.; Ega, T. K.; Paterno, L. G.; Pašti, I. A.; Kanoun, O. Versatile sensing capabilities of layer-by-layer deposited polyaniline-reduced graphene oxide composite-based sensors. *Sensors and Actuators B: Chemical* **2023**, *390*, 133988. DOI: 10.1016/j.snb.2023.133988.
(27) Tursunniyaz, M.; Agarwal, V.; Meredith, A.; Andrews, J. Hybrid nanomaterial inks for printed resistive temperature sensors with tunable properties to maximize sensitivity. *Nanoscale* **2022**, *15* (1), 162–170. DOI: 10.1039/D2NR04005K. Published Online: Dec. 22, 2022.
(28) Phadkule, S. S.; Sarma, S. High-performance flexible temperature sensor from hybrid nanocomposite for continuous human body temperature monitoring. *Polymer Composites* **2023**, *44* (2), 1381–1391. DOI: 10.1002/pc.27178.

(29) Li, Q.; Bai, R.; Guo, L.; Gao, Y. All laser direct writing process for temperature sensor based on graphene and silver. *Frontiers of optoelectronics* **2024**, *17* (1), 5. DOI: 10.1007/s12200-024-00108-4. Published Online: Feb. 5, 2024.
(30) Kong, C.; Li, X.; Zhang, E.; Shi, J.; Ren, J.; Li, C.; Wang, H.; Wu, K. A fiber-shaped temperature sensor composed of chitosan/rGO with high sensitivity and ultra-fast response and recovery for real-time temperature monitoring. *Progress in Organic Coatings* **2024**, *186*, 107989. DOI: 10.1016/j.porgcoat.2023.107989.
